# Structural equation modeling with factors and composites within the framework of the basic design

**DOI:** 10.1007/s11634-025-00647-4

**Published:** 2025-09-08

**Authors:** Arthur Tenenhaus, Michel Tenenhaus, Theo K. Dijkstra

**Affiliations:** 1 CentraleSupélec, Laboratoire des Signaux et Systèmes, Université Paris-Saclay, Gif-sur-Yvette, France; 2 HEC Paris, Jouy-en-Josas, France; 3 Faculty of Economics and Business, University of Groningen, Groningen, The Netherlands

**Keywords:** Structural equation modeling, PLS-PM, PLSc, Composite model, Restricted maximum-likelihood

## Abstract

In this paper, we study structural equation modeling with factors and composites within the framework of the basic design. The PLS approach (i.e. PLS-SEM based on PLS-PM, PLSc and the composite model) used to estimate this model has many limitations, including lack of theoretical foundations and the use of iterative algorithms with no guaranteed convergence. To address these shortcomings, we introduce a novel method, SVD-SEM, which corrects these issues. Indeed, SVD-SEM relies on a non-iterative SVD-based algorithm for parameter estimation, producing consistent and asymptotically normal estimators, offering a statistically and computationally sound alternative to PLS-SEM. Additionally, we present the restricted maximum-likelihood approach (RML-SEM) for the basic design with factors and composites. SVD-SEM can serve as an initial solution for RML-SEM. To illustrate the performance of these methods, we discuss a Monte Carlo simulation on a nonrecursive model with factors and composites.

## Introduction

Structural equation modeling (SEM) is a comprehensive multivariate framework used to specify and examine a system of linear models involving both observed and latent variables. In SEM literature, a latent variable is often synonymous with a factor—an entity that is not directly measurable but exists independently of observed variables. Observed variables linked to a factor are called effect or reflective indicators. A latent variable can also be conceptualized as a weighted sum of observed variables, where the weights are unknown non-random terms. In this context the observed variables are referred to as composite indicators, and the weighted sum is called composite variable.

In this paper, we consider structural equation modeling with both factors and composites. We assume that the measurement model combines standard confirmatory factor analysis for the factors (Kline 2016) and the composite model for the composites (Dijkstra 2017; Schuberth 2017; Schamberger 2022). The structural equation model can be either recursive or nonrecursive. Some specific constraints for identification are assumed. This model, called “the basic design”, is particularly suitable for soft modeling where complex problems are rich in data but scarce in theoretical knowledge. The first proposal to address this situation was introduced by Wold (1980, 1982, 1985) with Partial Least Squares Path Modeling (PLS-PM). Note that the basic design of this paper deviates from Wold’s original formulation (Wold 1982) in an essential way: While Wold assumed all latent variables to be factors and considered only recursive structural equation models, we allow latent variables to be either factors or composites and consider both recursive and nonrecursive structural equation models.

The PLS algorithm proposed by Wold (1982) can be used for estimating the parameters of the basic design, but has some limitations. The PLS algorithm is a multiblock component method where block components -linear combinations of block indicators—serve as proxies for latent variables. For each block, the investigator must choose between two computational modes: Mode A (linked to principal component analysis) and Mode B (linked to canonical correlation analysis). Wold suggests selecting Mode A for reflective indicators (the latent variable determines the indicators) and Mode B for formative indicators (the indicators determine the latent variable). Hereafter, we list some properties of the PLS algorithm.

- When all LVs are factors and Mode A selected for all blocks, the PLS algorithm yields inconsistent estimators. However, these estimators can be corrected to recover consistency and asymptotic normality (CAN) as shown in Dijkstra (1981). This yields Consistent PLS (PLSc) detailed in Dijkstra and Henseler (2015). Nevertheless, in this situation, the PLS algorithm lacks a smooth optimality criterion (Krämer 2007), and its convergence is not theoretically guaranteed, although typically observed in practice.
- When Mode B is chosen for all blocks, the fixed-point equation of the PLS algorithm corresponds to the stationary equation of a correlation-based criterion. The algorithm converges under the Gauss–Seidel update, but convergence to the global optimum is not assured (Hanafi 2007; Krämer 2007; Tenenhaus & Tenenhaus 2011; Tenenhaus et al. 2017).
- In the framework of the composite model, the PLS algorithm, with Mode B selected for all blocks, produces CAN estimators (Dijkstra 2017).

The cSEM package (composite-based SEM), developed by Rademaker and Schuberth (2020), implements both PLSc and the composite model. This combination of methods is referred to as PLS-SEM in this paper, and should not be confused with PLS-PM. However, as the package relies on the PLS algorithm, it inherits its limitations.

To address these limitations, we introduce a novel, non-iterative estimation method called SVD-SEM. Through singular value decompositions, SVD-SEM yields CAN estimators of the parameters of the basic design. SVD-SEM aligns with the framework proposed by Dhaene & Rosseel (2023) on non-iterative estimators in the structural after measurement (SAM) approach to SEM. From these perspectives, SVD-SEM offers a sound statistically and computationally alternative to PLS-SEM.

Additionally, we consider the maximum-likelihood estimation method for the basic design. When the model only contains factors, the standard approach (Jöreskog 1978) applies. When the model contains composites, identification constraints are taken into account by using the restricted maximum likelihood approach (Silvey 1975). This method is referred to as RML-SEM in the remainder of the paper. We mention that an ML estimation of the composite model has already been investigated in Schamberger (2022). SVD-SEM estimates can be used as starting point for the iterative algorithm that produces the ML estimates.

The simplicity of the paper stems from the simple form of the basic design. However, approaches for SEM with factors and composites have been recently proposed for the general structural equation model: see Henseler (2021) and Schuberth (2021) for covariance-based SEM, and Hwang et al. (2021) for composite-based SEM.

The paper is organized as follows. We begin with a detailed description of the basic design. We establish that all parameters of this model can be explicitly expressed in terms of the covariance matrix of the observed variables. This leads to SVD-SEM. We show that CAN parameter estimators can be deduced from these relations. We discuss tests of significance, test-of-fit and single-block regression predictors of factors. Next, we introduce RML-SEM for the basic design. Then, we compare SVD-SEM and RML-SEM on a nonrecursive model with factors and composites in a simulation. Mathematical proof that SVD-SEM produces CAN estimators is given in the Appendix.

## The basic design for structural equation modeling with factors and composites

In this section, we describe in detail the basic design for structural equation modeling with factors and composites. The basic design is divided into two parts: the measurement model and the structural model, which are described hereafter.

### The measurement model

We consider a random column vector ${\boldsymbol{y}}$ of $p$ observed variables (also called indicators or manifest variables). The vector $\boldsymbol{y}$ is composed of $J$ subvectors (or blocks) ${\boldsymbol{y}}_{j}$, where $J \ge 2$. Each ${\boldsymbol{y}}_{j}$ contains $p_{j}$ indicators $y_{j1} ,...,y_{{jp_{j} }}$. A block component is any linear combination of $y_{j1} ,...,y_{{jp_{j} }}$. The vector of indicators ${\boldsymbol{y}}$ has zero mean and covariance matrix ${{\boldsymbol{\Sigma}}} = \left\{ {{{\boldsymbol{\Sigma}}}_{jk} } \right\}$, where ${{\boldsymbol{\Sigma}}}_{jk} = E\left( {\boldsymbol{y}_{j} \boldsymbol{y}_{k}^{t} } \right).$ No constraints are assumed on the distribution of ${\boldsymbol{y}}$ apart from the existence of moments. The existence of second order moments guarantees consistency of the estimators to be developed below; the existence of fourth-order moments yields asymptotic normality.

Some notations and general assumptions used throughout this paper are listed below.

- We assume that there exists a zero mean and unit variance random variable $\xi_{j}$ (called latent variable (LV) and unobservable) associated with each block ${\boldsymbol{y}}_{j}$.
- We denote by ${{\boldsymbol{\uplambda}}}_{j}^{{}} = \left( {cov\left( {y_{j1} ,\xi_{j} } \right);...;cov\left( {y_{{jp_{j} }} ,\xi_{j} } \right)} \right)$ the loading vector related to $\xi_{j}$. The semi-colon is used in this paper for vertical concatenation. Possible sign indeterminacy is removed by imposing the sign constraint $\lambda_{j1} = cov\left( {y_{j1} ,\xi_{j} } \right)> 0$.
- The correlation matrix ${\mathbf{R}} = \left\{ {\rho_{jk} } \right\}$ of $\xi_{1} ,...,\xi_{J}$ is assumed to be positive definite.
- Each intra-block covariance matrix ${{\boldsymbol{\Sigma}}}_{jj}$ is assumed to be positive definite.

The basic design for factors and composites relies on Wold’s fundamental principle of soft modeling: all information between blocks is conveyed solely by the LVs $\xi_{j}$ through formula

1 $$cov\left( {y_{jh} ,y_{k\ell } } \right) = cor\left( {\xi_{j} ,\xi_{k} } \right)cov\left( {y_{jh} ,\xi_{j} } \right)cov\left( {y_{k\ell } ,\xi_{k} } \right).$$

This formula is written more compactly through the rank one covariance matrix

2 $${{\boldsymbol{\Sigma}}}_{jk} = \rho_{jk} {{\boldsymbol{\uplambda}}}_{j} {{\boldsymbol{\uplambda}}}_{k}^{t}.$$

We now describe the two specific situations studied in this paper, according to the nature of the relationship between the block ${\boldsymbol{y}}_{j}$ and its related LV $\xi_{j}$.

#### Reflective block and factor

A block-vector ${\boldsymbol{y}}_{j}$ is called reflective when the observed variables $y_{j1} ,...,y_{{jp_{j} }}$ are effect indicators depending on the LV $\xi_{j}$. We assume $p_{j} \ge 2$. The case of a single effect indicator is discussed in Kline (2016, pp. 214–217). In the framework of this paper, a block with a single indicator is considered as formative (see below). The LV $\xi_{j}$ determines ${\boldsymbol{y}}_{j}$ up to measurement errors:

3 $$\boldsymbol{y}_{j} = {{\boldsymbol{\uplambda}}}_{j} \xi_{j} + {\boldsymbol{\varepsilon} }_{j} ,$$

where ${\boldsymbol{\varepsilon}}_{j} = \left( {\varepsilon_{j1} ;...;\varepsilon_{{jp_{j} }} } \right)$ is a vector of measurement errors. We assume that all errors are mutually uncorrelated, and uncorrelated with all unobservable variables and all composite indicators. Therefore, $\theta_{jh} = var\left( {\varepsilon_{jh} } \right) = var\left( {y_{jh} } \right) - \lambda_{jh}^{2}$. Let ${{\boldsymbol{\Theta}}}_{j} = E\left( {{\boldsymbol{\varepsilon}}_{j} {\boldsymbol{\varepsilon}}_{j}^{t} } \right) = {\mathrm{diag}}\left( {\theta_{j1} ,...,\theta_{{jp_{j} }} } \right)$. The above assumptions imply that (2) is satisfied and that the covariance matrix ${{\boldsymbol{\Sigma}}}_{jj}$ of ${\boldsymbol{y}}_{j}$ can be written as

4 $${{\boldsymbol{\Sigma}}}_{jj} = E\left( {\boldsymbol{y}_{j} \boldsymbol{y}_{j}^{t} } \right) = {{\boldsymbol{\uplambda}}}_{j} {{\boldsymbol{\uplambda}}}_{j}^{t} + {{\boldsymbol{\Theta}}}_{j} .$$

The LV $\xi_{j}$ associated with a reflective block is referred to as factor in this paper.

#### Formative block and composite

A block-vector ${\boldsymbol{y}}_{j}$ is called formative when the LV $\xi_{j}$ is completely determined by the observed variables $y_{j1} ,...,y_{{jp_{j} }}$ and is equal to ${\mathbf{w}}_{j}^{t} \boldsymbol{y}_{j}$. The weight-vector ${\mathbf{w}}_{j}$ contains $p_{j}$ non-random unknown elements. The observed variables $y_{j1} ,...,y_{{jp_{j} }}$ are called composite indicators and $\xi_{j}$ composite (Grace and Bollen 2008). In the composite model of Dijkstra (2017), the composites $\xi_{j}$ satisfy the Wold’s fundamental principle of soft modeling. A formative block may contain a single variable $y_{j}$ (for example a covariate). In that case, the related composite is this single variable standardized and Formula (1) is assumed to be satisfied. Thus, in this situation, (2) becomes ${{\boldsymbol{\Sigma}}}_{jk} = cov\left( {y_{j} ,{\boldsymbol{y}}_{k} } \right) = \rho_{jk} \sqrt {var\left( {y_{j} } \right)} {{\boldsymbol{\uplambda}}}_{k}^{t}$. The following identities will be used in the continuation of the paper:

5 $${\text{unit variance composite}}:\quad {\mathbf{w}}_{j}^{t} {{\boldsymbol{\Sigma}}}_{jj} {\mathbf{w}}_{j} = 1,$$

6 $${\mathrm{loading}}{:}\quad {{\boldsymbol{\uplambda}}}_{j}^{{}} = {{\boldsymbol{\Sigma}}}_{jj} {\mathbf{w}}_{j} ,$$

7 $${\text{deduced from }}\left( {5} \right){\text{ and }}\left( {6} \right){:}\quad {{\boldsymbol{\uplambda}}}_{j}^{t} {{\boldsymbol{\Sigma}}}_{jj}^{ - 1} {{\boldsymbol{\uplambda}}}_{j}^{{}} = 1.$$

#### Population covariance matrix implied by the measurement model of the basic design

The covariance matrix ${{\boldsymbol{\Sigma}}} = \left( {{{\boldsymbol{\Sigma}}}_{jk} } \right)$ of the whole set of observed variables implied by the measurement model of the basic design can be expressed as

8 $${{\boldsymbol{\Sigma}}} = \left[ {\begin{array}{*{20}c} {{\mathbf{\overset{\lower0.5em\hbox{$\smash{\scriptscriptstyle\frown}$}}{{\boldsymbol{\Sigma}}}}}_{11} } & {} & {} & {} & {} \\ {\rho_{21} {{\boldsymbol{\uplambda}}}_{2} {{\boldsymbol{\uplambda}}}_{1}^{t} } & {{\mathbf{\overset{\lower0.5em\hbox{$\smash{\scriptscriptstyle\frown}$}}{{\boldsymbol{\Sigma}}}}}_{22} } & {} & {} & {} \\ {\rho_{31} {{\boldsymbol{\uplambda}}}_{3} {{\boldsymbol{\uplambda}}}_{1}^{t} } & {\rho_{32} {{\boldsymbol{\uplambda}}}_{3} {{\boldsymbol{\uplambda}}}_{2}^{t} } & {{\mathbf{\overset{\lower0.5em\hbox{$\smash{\scriptscriptstyle\frown}$}}{{\boldsymbol{\Sigma}}}}}_{33} } & {} & {} \\ \vdots & \vdots & \vdots & \ddots & {} \\ {\rho_{J1} {{\boldsymbol{\uplambda}}}_{J} {{\boldsymbol{\uplambda}}}_{1}^{t} } & {\rho_{J2} {{\boldsymbol{\uplambda}}}_{J} {{\boldsymbol{\uplambda}}}_{2}^{t} } & {\rho_{J3} {{\boldsymbol{\uplambda}}}_{J} {{\boldsymbol{\uplambda}}}_{3}^{t} } & \cdots & {{\mathbf{\overset{\lower0.5em\hbox{$\smash{\scriptscriptstyle\frown}$}}{{\boldsymbol{\Sigma}}}}}_{JJ} } \\ \end{array} } \right],$$

where

9 $${\mathbf{\overset{\lower0.5em\hbox{$\smash{\scriptscriptstyle\frown}$}}{\boldsymbol{\Sigma}}}}_{jj} = \left\{ {\begin{array}{*{20}ll} {{{\boldsymbol{\uplambda}}}_{j} {{\boldsymbol{\uplambda}}}_{j}^{t} + {{\boldsymbol{\Theta}}}_{j} ,}&\quad {\text{for a reflective block,}} \\ {{{\boldsymbol{\Sigma}}}_{jj} {,}} &\quad {{\text{for a formative block}}{.}} \\ \end{array}} \right.$$

All of the assumptions mentioned above ensure that the measurement model is identified. This property is shown below by expressing the loading vectors ${{\boldsymbol{\uplambda}}}_{j}^{{}}$ and the correlations $\rho_{jk}$ in terms of elements of ${{\boldsymbol{\Sigma}}}$.

### Expressing the measurement model parameters in terms of elements of ${{\boldsymbol{\Sigma}}}$

The loading vector ${{\boldsymbol{\uplambda}}}_{j}$ can be expressed in terms of elements of ${{\boldsymbol{\Sigma}}}$ using a two-step procedure. The vector ${{\boldsymbol{\uplambda}}}_{j}$ can always be written as the product of two terms: ${{\boldsymbol{\uplambda}}}_{j} = \left(\boldsymbol{\uplambda}_{j} /\left\| {{{\boldsymbol{\uplambda}}}_{j} } \right\|\right) \cdot \left\| {{{\boldsymbol{\uplambda}}}_{j} } \right\|$. In the first step, we show that the normalized loading vector ${{\boldsymbol{\uplambda}}}_{j}^{*} = {{\boldsymbol{\uplambda}}}_{j} /\left\| {{{\boldsymbol{\uplambda}}}_{j} } \right\|$ is eigenvector of some matrix, function of the ${{\boldsymbol{\Sigma}}}_{jk}$’s for $k \ne j$. In the second step, $\left\| {{{\boldsymbol{\uplambda}}}_{j} } \right\|$ is expressed in terms of ${{\boldsymbol{\uplambda}}}_{j}^{*}$ and ${{\boldsymbol{\Sigma}}}_{jj}$; this expression depends upon the formative or reflective nature of the block.

#### *Computing the normalized loading vectors*${{\boldsymbol{\uplambda}}}_{j}^{*}$*in terms of elements of*${{\boldsymbol{\Sigma}}}$

The normalized loading vector ${{\boldsymbol{\uplambda}}}_{j}^{*} = {{\boldsymbol{\uplambda}}}_{j} /\left\| {{{\boldsymbol{\uplambda}}}_{j} } \right\|$ is the left singular vector of the rank one matrix ${{\boldsymbol{\Sigma}}}_{jk} = \rho_{jk} {{\boldsymbol{\uplambda}}}_{j} {{\boldsymbol{\uplambda}}}_{k}^{t}$ for any $k \ne j$ and such that $\lambda_{j1}^{*}> 0$. To avoid choosing a specific pair $k \ne j,$ we can choose ${{\boldsymbol{\uplambda}}}_{j}^{*}$ as the unit norm eigenvector of the rank one matrix $\sum\limits_{k = 1,k \ne j}^{J} {{{\boldsymbol{\Sigma}}}_{jk} } {{\boldsymbol{\Sigma}}}_{kj}$ corresponding to the nonzero eigenvalue.

#### *Computation of*$\left\| {{{\boldsymbol{\uplambda}}}_{j} } \right\|$*in terms of elements of*${{\boldsymbol{\Sigma}}}$

The computation of $\left\| {{{\boldsymbol{\uplambda}}}_{j} } \right\|$ in terms of ${{\boldsymbol{\uplambda}}}_{j}^{*}$ and ${{\boldsymbol{\Sigma}}}_{jj} = \left\{ {\sigma_{hk}^{j} } \right\}$ depends upon the nature of the block:

– For formative blocks, using (7), we obtain
  10 $$\left\| {{{\boldsymbol{\uplambda}}}_{j} } \right\|^{2} = 1/{{\boldsymbol{\uplambda}}}_{j}^{*t} {{\boldsymbol{\Sigma}}}_{jj}^{ - 1} {{\boldsymbol{\uplambda}}}_{j}^{*} .$$
– For reflective blocks, we deduce from (4), for $h \ne k$,
  11 $$\left\| {{{\boldsymbol{\uplambda}}}_{j} } \right\|^{2} = \frac{{\sigma_{hk}^{j} }}{{\lambda_{jh}^{*} \lambda_{jk}^{*} }}.$$

To avoid choosing a specific pair $h \ne k$, we consider the least squares estimate of the slope $\left\| {{{\boldsymbol{\uplambda}}}_{j} } \right\|^{2}$ in the regression line $\sigma_{hk}^{j} = \left\| {{{\boldsymbol{\uplambda}}}_{j} } \right\|^{2} \lambda_{jh}^{*} \lambda_{jk}^{*}$:

12 $$\left\| {{{\boldsymbol{\uplambda}}}_{j} } \right\|^{2} = \frac{1}{{\sum\limits_{h \ne k} {\left( {\lambda_{jh}^{*} \lambda_{jk}^{*} } \right)^{2} } }}\sum\limits_{h \ne k} {\lambda_{jh}^{*} \lambda_{jk}^{*} \sigma_{hk}^{j} } \, = \frac{{{{\boldsymbol{\uplambda}}}_{j}^{*t} \left( {{{\boldsymbol{\Sigma}}}_{jj} - diag\left( {{{\boldsymbol{\Sigma}}}_{jj} } \right)} \right){{\boldsymbol{\uplambda}}}_{j}^{*} }}{{1 - {{\boldsymbol{\uplambda}}}_{j}^{*t} diag\left( {{{\boldsymbol{\uplambda}}}_{j}^{*} {{\boldsymbol{\uplambda}}}_{j}^{*t} } \right){{\boldsymbol{\uplambda}}}_{j}^{*} }}.$$

We denote by $d_{j}^{{}}$ the expression of $\left\| {{{\boldsymbol{\uplambda}}}_{j} } \right\|$ for both situations, formative (10) and reflective (12).

#### *Expressing loading and weight vectors in terms of elements of*${{\boldsymbol{\Sigma}}}$

The loading vectors for all blocks are now expressed in terms of elements of ${{\boldsymbol{\Sigma}}}$:

13 $${{\boldsymbol{\uplambda}}}_{j} = d_{j}^{{}} {{\boldsymbol{\uplambda}}}_{j}^{*} .$$

For formative blocks, using (6), we obtain:

14 $${\mathbf{w}}_{j} = {{\boldsymbol{\Sigma}}}_{jj}^{ - 1} {{\boldsymbol{\uplambda}}}_{j} = d_{j}^{{}} {{\boldsymbol{\Sigma}}}_{jj}^{ - 1} {{\boldsymbol{\uplambda}}}_{j}^{*} .$$

#### *Expressing the correlation*$\rho_{jk}$*in terms of elements of*${{\boldsymbol{\Sigma}}}$

We deduce from (2) the following expression for the correlation between LVs:

15 $$\rho_{jk} = \frac{{{{\boldsymbol{\uplambda}}}_{j}^{t} {{\boldsymbol{\Sigma}}}_{jk} {{\boldsymbol{\uplambda}}}_{k} }}{{\left\| {{{\boldsymbol{\uplambda}}}_{j} } \right\|^{2} \left\| {{{\boldsymbol{\uplambda}}}_{k} } \right\|^{2} }} = \frac{{{{\boldsymbol{\uplambda}}}_{j}^{*t} {{\boldsymbol{\Sigma}}}_{jk} {{\boldsymbol{\uplambda}}}_{k}^{*} }}{{d_{j} d_{k} }}.$$

Hence, the normalized loading vectors ${{\boldsymbol{\uplambda}}}_{1}^{*} ,...,{{\boldsymbol{\uplambda}}}_{J}^{*}$ play a central role in this paper because all parameters of the measurement model can be expressed in terms of ${{\boldsymbol{\uplambda}}}_{1}^{*} ,...,{{\boldsymbol{\uplambda}}}_{J}^{*}$ and elements of ${{\boldsymbol{\Sigma}}}$.

### Structural equations on factors and composites

We consider a situation where a set of simultaneous equations is satisfied by the LVs. We denote by ${\boldsymbol{\xi}} = \left( {\xi_{1} ;...;\xi_{J} } \right)$ the column vector of $J = n + m$ LVs. This vector is organized in such a way that it is partitioned into a vector ${\boldsymbol{\xi}}_{exo} = \left( {\xi_{1} ;...;\xi_{n} } \right)$ of *n* exogenous LVs and a vector ${\boldsymbol{\xi}}_{endo} = \left( {\xi_{n + 1} ;...;\xi_{n + m} } \right)$ of *m* endogenous LVs. The model is written as

16 $${\boldsymbol{\xi}}_{endo} = {\mathbf{B}}{\boldsymbol{\xi} }_{endo} + {{\boldsymbol{\Gamma}}}{\boldsymbol{\xi} }_{exo} + {\boldsymbol{\zeta} ,}$$

where ${\mathbf{B}}$ and ${{\boldsymbol{\Gamma}}}$ are matrices of path coefficients. The diagonal of ${\mathbf{B}}$ is null and the matrix ${\mathbf{I}} - {\mathbf{B}}$ is supposed to be invertible. The disturbance vector ${\boldsymbol{\zeta} } = \left( {\zeta_{1} ;...;\zeta_{m} } \right)$ has zero mean, covariance matrix ${{\boldsymbol{\Psi}}} = E\left( {{\boldsymbol{\zeta} \boldsymbol{\zeta} }^{t} } \right)$ and is assumed to be uncorrelated with the vector ${\boldsymbol{\xi} }_{exo}$ of exogenous LVs. The correlation matrices of the exogenous and endogenous LVs are denoted as ${{\boldsymbol{\Phi}}}_{exo} = E\left( {{\boldsymbol{\xi} }_{exo} {\boldsymbol{\xi} }_{exo}^{t} } \right)$ and ${{\boldsymbol{\Phi}}}_{endo} = E\left( {{\boldsymbol{\xi} }_{endo} {\boldsymbol{\xi} }_{endo}^{t} } \right)$.

Consider the reduced form of (16):

17 $${\boldsymbol{\xi} }_{endo} = \left( {{\mathbf{I}} - {\mathbf{B}}} \right)^{ - 1} {{\boldsymbol{\Gamma}}}{\boldsymbol{\xi} }_{exo} + \left( {{\mathbf{I}} - {\mathbf{B}}} \right)^{ - 1} {\boldsymbol{\zeta} }{.}$$

We deduce from (17) the usual formulas:

18 $${{\boldsymbol{\Phi}}}_{endo} = \left( {{\mathbf{I}} - {\mathbf{B}}} \right)^{ - 1} \left( {{\boldsymbol{\Gamma \Phi }}_{exo} {{\boldsymbol{\Gamma}}}^{t} + {{\boldsymbol{\Psi}}}} \right)\left( {{\mathbf{I}} - {\mathbf{B}}^{t} } \right)^{ - 1} ,$$

19 $$E\left( {{\boldsymbol{\xi} }_{endo} {\boldsymbol{\xi} }_{exo}^{t} } \right) = \left( {{\mathbf{I}} - {\mathbf{B}}} \right)^{ - 1} {\boldsymbol{\Gamma \Phi }}_{exo}^{{}} .$$

Model (16) is assumed to be identified. This means that there is unicity of the decomposition (19). More precisely, this means that if ${\mathbf{B}}_{1} ,{{\boldsymbol{\Gamma}}}_{1}$ and ${\mathbf{B}}_{2} ,{{\boldsymbol{\Gamma}}}_{2}$ satisfy (19), then ${\mathbf{B}}_{1} = {\mathbf{B}}_{2}$ and ${{\boldsymbol{\Gamma}}}_{1} = {{\boldsymbol{\Gamma}}}_{2}$. Parameters ${\mathbf{B}}$, ${{\boldsymbol{\Gamma}}}$ and ${{\boldsymbol{\Psi}}}$ of identified models must satisfy some conditions. These conditions depend on the type of structural regression model: recursive or nonrecursive. A model is recursive when two conditions are satisfied: ${\mathbf{B}}$ can be written as a lower triangular matrix and ${{\boldsymbol{\Psi}}}$ is diagonal. Recursive models are always identified. For nonrecursive models, identification is obtained by imposing some restrictions on ${\mathbf{B}}$ and ${{\boldsymbol{\Gamma}}}$. The most common restrictions consist in cancelling some elements of ${\mathbf{B}}$ and ${{\boldsymbol{\Gamma}}}$. A necessary condition for identification of nonrecursive models is the order condition: at least $m - 1$ explanatory variables are excluded from each equation in (16). Moreover, we assume that ${{\boldsymbol{\Psi}}}$ contains no restrictions, apart the constraint in (18) that ${{\boldsymbol{\Phi}}}_{endo}$ is a correlation matrix. For more details on the identification problem, see Johnston (1972) and Bollen (1989).

#### *Expressing *${\mathbf{B}}$*and *${{\boldsymbol{\Gamma}}}$* in terms of elements of *${\mathbf{R}}$

Matrices ${\mathbf{B}}$ and ${{\boldsymbol{\Gamma}}}$ can be expressed in terms of elements of ${\mathbf{R}} = \left\{ {\rho_{jk} } \right\}$. We consider separately each equation in (16). We denote by ${{\boldsymbol{\upbeta}}}_{i}^{t}$ and ${{\boldsymbol{\upgamma}}}_{i}^{t}$ the *i*th rows of, respectively, ${\mathbf{B}}$ and ${{\boldsymbol{\Gamma}}}$, removing all zero values. Let ${\boldsymbol{\xi} }_{endo,i}$ be the column vector of endogenous LVs corresponding to the coefficients of ${{\boldsymbol{\upbeta}}}_{i}^{{}}$, ${\boldsymbol{\xi}}_{exo,i}$ the column vector of exogenous LVs corresponding to the coefficients of ${{\boldsymbol{\upgamma}}}_{i}^{{}}$ and ${\boldsymbol{\xi} }_{i} = \left( {{\boldsymbol{\xi} }_{endo,i} ;{\boldsymbol{\xi} }_{exo,i} } \right)$. Using these notations, the *i*th structural equation in (16) can be written as

20 $$\xi_{n + i} = {{\boldsymbol{\upbeta}}}_{i}^{t} {\boldsymbol{\xi} }_{endo,i} + {{\boldsymbol{\upgamma}}}_{i}^{t} {\boldsymbol{\xi} }_{exo,i} + \zeta_{i} .$$

Recursive models have the property that the disturbance $\zeta_{i}$ is uncorrelated with ${\boldsymbol{\xi} }_{i} = \left( {{\boldsymbol{\xi} }_{endo,i} ;{\boldsymbol{\xi} }_{exo,i} } \right)$. Using straightforward algebra, the following formulas, similar to OLS and 2SLS formulas, are obtained:

-When the disturbance $\zeta_{i}$ is uncorrelated with ${\boldsymbol{\xi} }_{i}$:

21 $$\left( {\begin{array}{*{20}c} {{{\boldsymbol{\upbeta}}}_{i} } \\ {{{\boldsymbol{\upgamma}}}_{i} } \\ \end{array} } \right) = E\left( {{\boldsymbol{\xi} }_{i} {\boldsymbol{\xi} }_{i}^{t} } \right)^{ - 1} E\left( {{\boldsymbol{\xi} }_{i} \xi_{n + i} } \right).$$

-When the disturbance $\zeta_{i}$ is correlated with some endogenous variables in ${\boldsymbol{\xi} }_{endo,i}$:

22 $$\begin{aligned} \left( {\begin{array}{*{20}c} {{{\boldsymbol{\upbeta}}}_{i} } \\ {{{\boldsymbol{\upgamma}}}_{i} } \\ \end{array} } \right) & = \left( {\begin{array}{*{20}c} {E\left( {{\boldsymbol{\xi} }_{endo,i} {\boldsymbol{\xi} }_{exo}^{t} } \right){{\boldsymbol{\Phi}}}_{exo}^{ - 1} E\left( {{\boldsymbol{\xi} }_{exo} {\boldsymbol{\xi} }_{endo,i}^{t} } \right)} & {E\left( {{\boldsymbol{\xi} }_{endo,i} {\boldsymbol{\xi} }_{exo,i}^{t} } \right)} \\ {E\left( {{\boldsymbol{\xi} }_{exo,i} {\boldsymbol{\xi} }_{endo,i}^{t} } \right)} & {E\left( {{\boldsymbol{\xi} }_{exo,i} {\boldsymbol{\xi} }_{exo,i}^{t} } \right)} \\ \end{array} } \right)^{ - 1} \\ & \quad \times \left( {\begin{array}{*{20}c} {E\left( {{\boldsymbol{\xi} }_{endo,i} {\boldsymbol{\xi} }_{exo}^{t} } \right){{\boldsymbol{\Phi}}}_{exo}^{ - 1} E\left( {{\boldsymbol{\xi} }_{exo} \xi_{n + i} } \right)} \\ {E\left( {{\boldsymbol{\xi} }_{exo,i} \xi_{n + i} } \right)} \\ \end{array} } \right) \cdot \\ \end{aligned}$$

The matrices ${\mathbf{B}}$ and ${{\boldsymbol{\Gamma}}}$ are then derived by putting back the previously removed zero values. Furthermore, as the model (16) is assumed to be identified, they are the unique solution of (19).

#### Model-implied correlation matrix for LVs

We study separately the correlation matrix ${\mathbf{R}} = \left\{ {\rho_{jk} } \right\}$ for nonrecursive and recursive models. The correlation matrix ${{\boldsymbol{\Phi}}}_{endo}$ is considered as a free parameter in nonrecursive models and redundant in recursive ones. The covariance matrix ${{\boldsymbol{\Psi}}} = \left\{ {\psi_{ij} } \right\}$ of the disturbance ${\boldsymbol{\zeta} }$ is a redundant parameter in both situations.

##### Nonrecursive model

We deduce from (18) the expression of ${{\boldsymbol{\Psi}}}$ in terms of the other parameters:

23 $${{\boldsymbol{\Psi}}} = \left( {{\mathbf{I}} - {\mathbf{B}}} \right){{\boldsymbol{\Phi}}}_{endo} \left( {{\mathbf{I}} - {\mathbf{B}}^{t} } \right) - {\boldsymbol{\Gamma \Phi }}_{exo} {{\boldsymbol{\Gamma}}}^{t} .$$

In the usual presentation of structural equation models with observed variables, ${{\boldsymbol{\Psi}}}$ is free and formula (18) is used to compute the covariance matrix of the endogenous variables. Here, due to the unit variance constraint on the LVs, it’s simpler to consider the opposite situation: ${{\boldsymbol{\Phi}}}_{endo}$ is free and ${{\boldsymbol{\Psi}}}$ is a redundant parameter given by formula (23). Hence, the correlation matrix ${\mathbf{R}} = \left\{ {\rho_{jk} } \right\}$ for a nonrecursive model is written as:

24 $${\mathbf{R}} = \left[ {\begin{array}{*{20}c} {{{\boldsymbol{\Phi}}}_{exo} } & {{{\boldsymbol{\Phi}}}_{exo} {{\boldsymbol{\Gamma}}}^{t} \left( {{\mathbf{I}} - {\mathbf{B}}^{t} } \right)^{ - 1} } \\ {\left( {{\mathbf{I}} - {\mathbf{B}}} \right)^{ - 1} {\boldsymbol{\Gamma \Phi }}_{exo} } & {{{\boldsymbol{\Phi}}}_{endo} } \\ \end{array} } \right].$$

##### Recursive model

In recursive models, the matrix ${{\boldsymbol{\Psi}}}$ is diagonal. Furthermore, as the correlation matrix ${{\boldsymbol{\Phi}}}_{endo}$ satisfies (18), ${{\boldsymbol{\Psi}}}$ satisfies the constraint

25 $${\mathrm{diag}}\left[ {\left( {{\mathbf{I}} - {\mathbf{B}}} \right)^{ - 1} \left( {{\boldsymbol{\Gamma \Phi }}_{exo} {{\boldsymbol{\Gamma}}}^{t} + {{\boldsymbol{\Psi}}}} \right)\left( {{\mathbf{I}} - {\mathbf{B}}^{t} } \right)^{ - 1} } \right] = {\mathbf{I}}.$$

Note ${\mathrm{D}}\left( {\mathbf{M}} \right)$ the vector formed by the diagonal of the matrix **M**. Let ${{\boldsymbol{\uppsi}}} = {\mathrm{D}}\left( {{\boldsymbol{\Psi}}} \right)$. Using the Hadamard product we get the identity

26 $$\left( {{\mathbf{I}} - {\mathbf{B}}} \right)^{ - 1} \odot \left( {{\mathbf{I}} - {\mathbf{B}}} \right)^{ - 1} {{\boldsymbol{\uppsi}}} = {\mathrm{D}}\left[ {\left( {{\mathbf{I}} - {\mathbf{B}}} \right)^{ - 1} {{\boldsymbol{\Psi}}}\left( {{\mathbf{I}} - {\mathbf{B}}^{t} } \right)^{ - 1} } \right].$$

Therefore, we deduce from (25):

27 $${{\boldsymbol{\uppsi}}} = \left[ {\left( {{\mathbf{I}} - {\mathbf{B}}} \right)^{ - 1} \odot \left( {{\mathbf{I}} - {\mathbf{B}}} \right)^{ - 1} } \right]^{ - 1} {\mathrm{D}}\left[ {{\mathbf{I}} - \left( {{\mathbf{I}} - {\mathbf{B}}} \right)^{ - 1} {\boldsymbol{\Gamma \Phi }}_{exo} {{\boldsymbol{\Gamma}}}^{t} \left( {{\mathbf{I}} - {\mathbf{B}}^{t} } \right)^{ - 1} } \right].$$

Then, the model-implied correlation matrix ${\mathbf{R}} = \left\{ {\rho_{jk} } \right\}$ for a recursive model is written as:

28 $${\mathbf{R}} = \left[ {\begin{array}{*{20}c} {{{\boldsymbol{\Phi}}}_{exo} } & {{{\boldsymbol{\Phi}}}_{exo} {{\boldsymbol{\Gamma}}}^{t} \left( {{\mathbf{I}} - {\mathbf{B}}^{t} } \right)^{ - 1} } \\ {\left( {{\mathbf{I}} - {\mathbf{B}}} \right)^{ - 1} {\boldsymbol{\Gamma \Phi }}_{exo} } & {\left( {{\mathbf{I}} - {\mathbf{B}}} \right)^{ - 1} \left( {{\boldsymbol{\Gamma \Phi }}_{exo} {{\boldsymbol{\Gamma}}}^{t} + {{\boldsymbol{\Psi}}}} \right)\left( {{\mathbf{I}} - {\mathbf{B}}^{t} } \right)^{ - 1} } \\ \end{array} } \right],$$

where ${{\boldsymbol{\Psi}}}$ is a redundant parameter given by (27).

#### *Expressing*${\mathbf{B}}$, ${{\boldsymbol{\Gamma}}}$,${{\boldsymbol{\Psi}}}$*in terms of elements of*${{\boldsymbol{\Sigma}}}$

Parameters ${\mathbf{B}}$, ${{\boldsymbol{\Gamma}}}$ and ${{\boldsymbol{\Psi}}}$ of the structural equation model (16) have been expressed in terms of elements of ${\mathbf{R}} = \left\{ {\rho_{jk} } \right\}$. According to formula (15), this correlation matrix ${\mathbf{R}}$ can be expressed in terms of ${{\boldsymbol{\uplambda}}}_{1}^{*} ,...,{{\boldsymbol{\uplambda}}}_{J}^{*}$ and elements of ${{\boldsymbol{\Sigma}}}$. Therefore, ${\mathbf{B}}$, ${{\boldsymbol{\Gamma}}}$, ${{\boldsymbol{\Psi}}}$ can also be expressed in terms of ${{\boldsymbol{\uplambda}}}_{1}^{*} ,...,{{\boldsymbol{\uplambda}}}_{J}^{*}$ and elements of ${{\boldsymbol{\Sigma}}}$, and, consequently, in terms of elements of ${{\boldsymbol{\Sigma}}}$.

#### *Coefficient of determination *$R_{i}^{2}$

For all models (recursive or nonrecursive), the coefficient of determination $R_{i}^{2}$ for the unit variance endogenous variable $\xi_{n + i}$ explained by the LVs in Eq. (20) is computed as

29 $$R_{i}^{2} = 1 - \psi_{ii} .$$

### Model implied covariance matrix for the basic design

Let ${{\boldsymbol{\Lambda}}} = {\mathrm{diag}}\left( {{{\boldsymbol{\uplambda}}}_{1} ,...,{{\boldsymbol{\uplambda}}}_{J} } \right)$ and ${{\boldsymbol{\Theta}}} = {\mathrm{diag}}\left( {{{\boldsymbol{\Theta}}}_{1} ,...,{{\boldsymbol{\Theta}}}_{J} } \right)$. The columns of these matrices are organized as the vector of LVs ${\boldsymbol{\xi} } = \left( {{\boldsymbol{\xi} }_{exo} ;{\boldsymbol{\xi} }_{endo} } \right)$. Let ${{\boldsymbol{\uptheta}}}$ be the vector of all model parameters. Then, the model-implied covariance matrix of the structural equation model with factors and composites in the framework of the basic design is obtained by inserting the model-implied correlation matrix ${\mathbf{R}}\left( {{\boldsymbol{\uptheta}}} \right)$ given in (24) or (28) into (8):

30 $${{\boldsymbol{\Sigma}}}\left( {{\boldsymbol{\uptheta}}} \right) = {\boldsymbol{\Lambda} \mathbf{R}}\left( {{\boldsymbol{\uptheta}}} \right){{\boldsymbol{\Lambda}}}^{t} + {{\boldsymbol{\Theta}}} + {\text{ diag}}\left[ {{{\boldsymbol{\Sigma}}}_{11} - \left( {{{\boldsymbol{\uplambda}}}_{1} {{\boldsymbol{\uplambda}}}_{1}^{t} + {{\boldsymbol{\Theta}}}_{1} } \right),...,{{\boldsymbol{\Sigma}}}_{JJ} - \left( {{{\boldsymbol{\uplambda}}}_{J} {{\boldsymbol{\uplambda}}}_{J}^{t} + {{\boldsymbol{\Theta}}}_{J} } \right)} \right].$$

**Fig. 1:**
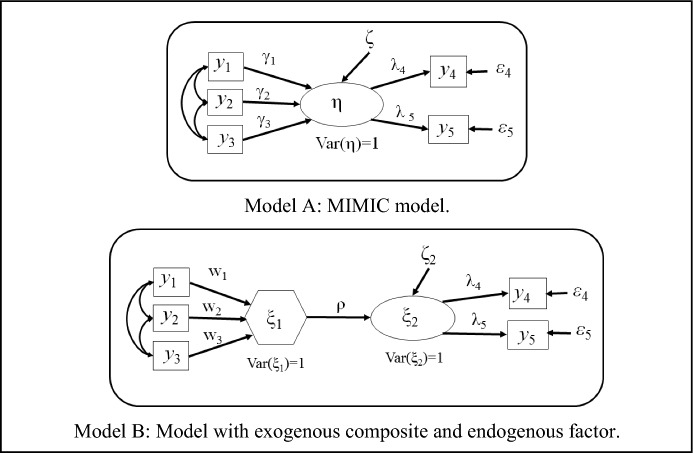
Equivalence between two models

We illustrate this section by considering the Multiple Indicators Multiple Causes (MIMIC) model, Model A in Fig. 1. The disturbance $\zeta$ and the measurement errors $\varepsilon_{4} ,\varepsilon_{5}$ are assumed to be mutually uncorrelated and uncorrelated with the causal indicators $y_{1} ,y_{2} ,y_{3}$. We now consider Model B with the same assumptions on the disturbance $\zeta_{2}$ and the measurement errors $\varepsilon_{4} ,\varepsilon_{5}$ as in Model A. The formative block is represented by a hexagon (Grace and Bollen (2008) introduced this notation). Computing the covariance matrices implied by these two models, we obtain:

$$\begin{aligned} {{\boldsymbol{\Sigma}}}\,\left( {\text{Model A}} \right) = \left[ {\begin{array}{*{20}c} {{{\boldsymbol{\Sigma}}}_{11} } & {{{\boldsymbol{\Sigma}}}_{11} {\boldsymbol{\gamma}} {{\boldsymbol{\uplambda}}}_{2}^{t} } \\ { {{\boldsymbol{\uplambda}}}_{2} {\boldsymbol{\gamma}}^{t} {{\boldsymbol{\Sigma}}}_{11} } & {{{\boldsymbol{\uplambda}}}_{2} {{\boldsymbol{\uplambda}}}_{2}^{t} + {{\boldsymbol{\Theta}}}_{2} } \\ \end{array} } \right]. \end{aligned}$$

and

$$\begin{aligned} {{\boldsymbol{\Sigma}}}\,\left( {\text{Model B}} \right) = \left[ {\begin{array}{*{20}c} {{{\boldsymbol{\Sigma}}}_{11} } & {\rho {{\boldsymbol{\Sigma}}}_{11} {\mathbf{w}}_{1}^{{}} {{\boldsymbol{\uplambda}}}_{2}^{t} } \\ {\rho {{\boldsymbol{\uplambda}}}_{2} {\mathbf{w}}_{1}^{t} {{\boldsymbol{\Sigma}}}_{11} } & {{{\boldsymbol{\uplambda}}}_{2} {{\boldsymbol{\uplambda}}}_{2}^{t} + {{\boldsymbol{\Theta}}}_{2} } \\ \end{array} } \right].  \end{aligned}$$

Therefore, both models are mathematically equivalent if ${\mathbf{w}}_{1} = {{\boldsymbol{\upgamma}}}/\rho$. Note that, as ${{\boldsymbol{\uplambda}}}_{1} = {{\boldsymbol{\Sigma}}}_{11} {\mathbf{w}}_{1}$, we recover the assumed model-implied covariance matrix corresponding to the connection between a formative block and a reflective one in the framework of the basic design. For Model B, ${{\boldsymbol{\Theta}}}_{1} = {\mathbf{0}}$, ${{\boldsymbol{\Sigma}}}_{22} = {{\boldsymbol{\uplambda}}}_{2} {{\boldsymbol{\uplambda}}}_{2}^{t} + {{\boldsymbol{\Theta}}}_{2}$ and the following decomposition is obtained:

$$\left[ {\begin{array}{*{20}c} {{{\boldsymbol{\Sigma}}}_{11} } & {\rho {{\boldsymbol{\uplambda}}}_{1} {{\boldsymbol{\uplambda}}}_{2}^{t} } \\ {\rho {{\boldsymbol{\uplambda}}}_{2} {{\boldsymbol{\uplambda}}}_{1}^{t} } & {{{\boldsymbol{\uplambda}}}_{2} {{\boldsymbol{\uplambda}}}_{2}^{t} + {{\boldsymbol{\Theta}}}_{2} } \\ \end{array} } \right] = \left[ {\begin{array}{*{20}c} {{{\boldsymbol{\uplambda}}}_{1} } & {\mathbf{0}} \\ {\mathbf{0}} & {{{\boldsymbol{\uplambda}}}_{2} } \\ \end{array} } \right]\left[ {\begin{array}{*{20}c} 1 & \rho \\ \rho & 1 \\ \end{array} } \right]\left[ {\begin{array}{*{20}c} {{{\boldsymbol{\uplambda}}}_{1}^{t} } & {\mathbf{0}} \\ {\mathbf{0}} & {{{\boldsymbol{\uplambda}}}_{2}^{t} } \\ \end{array} } \right] + \left[ {\begin{array}{*{20}c} {\mathbf{0}} & {\mathbf{0}} \\ {\mathbf{0}} & {{{\boldsymbol{\Theta}}}_{2} } \\ \end{array} } \right] + \left[ {\begin{array}{*{20}c} {{{\boldsymbol{\Sigma}}}_{11} - {{\boldsymbol{\uplambda}}}_{1} {{\boldsymbol{\uplambda}}}_{1}^{t} } & {\mathbf{0}} \\ {\mathbf{0}} & {\mathbf{0}} \\ \end{array} } \right].$$

## SVD-SEM

In the previous sections, we have found an explicit solution ${{\boldsymbol{\uptheta}}} = {\mathbf{f}}\left( {{\boldsymbol{\Sigma}}} \right)$ of the equation ${{\boldsymbol{\Sigma}}} = {{\boldsymbol{\Sigma}}}\left( {{\boldsymbol{\uptheta}}} \right)$ where ${{\boldsymbol{\Sigma}}}\left( {{\boldsymbol{\uptheta}}} \right)$ is given by (30). We have shown that the normalized loading vectors ${{\boldsymbol{\uplambda}}}_{1}^{*} ,...,{{\boldsymbol{\uplambda}}}_{J}^{*}$ were functions of ${{\boldsymbol{\Sigma}}}$, and ${{\boldsymbol{\uptheta}}}$ function of ${{\boldsymbol{\uplambda}}}_{1}^{*} ,...,{{\boldsymbol{\uplambda}}}_{J}^{*}$ and ${{\boldsymbol{\Sigma}}}$. Consequently, the estimator ${\hat{\boldsymbol{\theta }}}$ is simply obtained by using these functions with ${{\boldsymbol{\Sigma}}} = \left( {{{\boldsymbol{\Sigma}}}_{jk} } \right)$ replaced by its usual unbiased estimator ${\mathbf{S}} = \left( {{\mathbf{S}}_{jk} } \right)$: ${\hat{\boldsymbol{\theta }}} = {\mathbf{f}}\left( {\mathbf{S}} \right)$.

### Estimation of ${{\boldsymbol{\uplambda}}}_{j}^{*}$

Under the weak assumption that the vector ${\boldsymbol{y}}$ of the whole set of indicators has finite moment of order 4, ${\mathbf{S}}$ is a CAN estimator of ${{\boldsymbol{\Sigma}}}$. Let ${{\boldsymbol{\uplambda}}}_{j}^{*} \left( {\mathbf{S}} \right)$ be the unit norm eigenvector of $\, {\mathbf{A}}_{j} \left( {\mathbf{S}} \right) = \sum\limits_{k = 1,k \ne j}^{J} {{\mathbf{S}}_{jk} {\mathbf{S}}_{kj} }$, with $\lambda_{j1}^{*} \left( {\mathbf{S}} \right)> 0$, corresponding to the largest eigenvalue. We assume that this eigenvalue is simple. This is always verified in practical applications. This assumption implies that ${{\boldsymbol{\uplambda}}}_{j}^{*} \left( {\mathbf{S}} \right)$ is a continuous function of ${\mathbf{S}}$ (see Ortega 1990, p. 45). Therefore, ${{\boldsymbol{\uplambda}}}_{j}^{*} \left( {\mathbf{S}} \right)$ is a consistent estimator of ${{\boldsymbol{\uplambda}}}_{j}^{*} \left( {{\boldsymbol{\Sigma}}} \right) = {{\boldsymbol{\uplambda}}}_{j}^{*}$. Moreover, we show in Appendix that ${\mathbf{g}}\left( {{{\boldsymbol{\uplambda}}}_{1}^{*} \left( {\mathbf{S}} \right),...,{{\boldsymbol{\uplambda}}}_{J}^{*} \left( {\mathbf{S}} \right),{\mathbf{S}}} \right)$ is a CAN estimator of ${\mathbf{g}}\left( {{{\boldsymbol{\uplambda}}}_{1}^{*} ,...,{{\boldsymbol{\uplambda}}}_{J}^{*} ,{{\boldsymbol{\Sigma}}}} \right)$ for any smooth function **g**.

### CAN estimators of the parameters of the structural equation model with factors and composites for the basic design

We deduce from the previous results that CAN estimators of the various SEM parameters can be obtained by replacing ${{\boldsymbol{\Sigma}}}$ by ${\mathbf{S}}$ and ${{\boldsymbol{\uplambda}}}_{j}^{*}$ by ${{\boldsymbol{\uplambda}}}_{j}^{*} \left( {\mathbf{S}} \right)$ in formulas given above at the population level. We propose the following noniterative algorithm:

**Step 1.** We compute the estimator $\hat{\boldsymbol{\uplambda}}_{j}^{*} = {{\boldsymbol{\uplambda}}}_{j}^{*} \left( {\mathbf{S}} \right)$ of ${{\boldsymbol{\uplambda}}}_{j}^{*}$, as first eigenvector of $\, {\mathbf{A}}_{j} \left( {\mathbf{S}} \right)$ with $\lambda_{j1}^{*} \left( {\mathbf{S}} \right)> 0$.

**Step 2.** We compute the estimator $\hat{d}_{j}$ of $d_{j}$:

31 $$\displaystyle \hat{d}_{j} = \left\{ {\begin{array}{*{20}c} {\displaystyle\frac{1}{{\sqrt {\hat{\boldsymbol{\uplambda}}_{j}^{*t} {\mathbf{S}}_{jj}^{ - 1} {\hat{\boldsymbol{\uplambda}}}_{j}^{*} } }}} & {\text{for a formative block,}} \\\\ {\displaystyle \sqrt {\frac{{{\hat{\boldsymbol{\uplambda }}}_{j}^{*t} \left( {{\mathbf{S}}_{jj} - diag\left( {{\mathbf{S}}_{jj} } \right)} \right){\hat{\boldsymbol{\uplambda }}}_{j}^{*} }}{{1 - {\hat{\boldsymbol{\uplambda }}}_{j}^{*t} diag\left( {{\hat{\boldsymbol{\uplambda }}}_{j}^{*} {\hat{\boldsymbol{\uplambda }}}_{j}^{*t} } \right){\hat{\boldsymbol{\uplambda }}}_{j}^{*} }}} } & {{\text{for a reflective block}}{.}} \\ \end{array} } \right.$$

**Step 3.** CAN estimators of the measurement model parameters are obtained:

32 $${\hat{\boldsymbol{\uplambda }}}_{j} = \hat{d}_{j} {\hat{\boldsymbol{\uplambda }}}_{j}^{*} ,$$

33 $${\hat{\mathbf{w}}}_{j} = {\mathbf{S}}_{jj}^{ - 1} {\hat{\boldsymbol{\uplambda }}}_{j} \quad {\text{for a formative block}},$$

34 $${\hat{\boldsymbol{\Theta }}}_{j} = diag\left( {\hat{\theta }_{j1} ,...,\hat{\theta }_{{jp_{j} }} } \right),{\text{ where}}\quad \hat{\theta }_{jh} = s_{hh}^{j} - \hat{\lambda }_{jh}^{2} ,{\text{ for a reflective block}},$$

35 $$\displaystyle \tilde{\rho }_{jk} = \frac{{{\hat{\boldsymbol{\uplambda }}}_{j}^{*t} {\mathbf{S}}_{jk} {\hat{\boldsymbol{\uplambda }}}_{k}^{*} }}{{\hat{d}_{j} \hat{d}_{k} }},$$

where the tilde indicates a first estimator which does not consider the structural equation model.

**Step 4.** Using $\tilde{\rho }_{jk}$ instead of $\rho_{jk}$ in ${{\boldsymbol{\Phi}}}_{exo}$, ${{\boldsymbol{\Phi}}}_{endo}$, ${\mathbf{B}}$ and ${{\boldsymbol{\Gamma}}}$, CAN estimators ${\hat{\boldsymbol{\Phi }}}_{exo}$, ${\tilde{\boldsymbol{\Phi }}}_{endo}$, ${\hat{\mathbf{B}}}$ and ${\hat{\boldsymbol{\Gamma }}}$ are obtained. In the same way, a CAN estimator ${\hat{\boldsymbol{\Psi }}}$ of ${{\boldsymbol{\Psi}}}$ is obtained by using (23) for nonrecursive models and (27) for recursive ones. The final estimator ${\hat{\boldsymbol{\Phi }}}_{endo}$ is equal to ${\tilde{\boldsymbol{\Phi }}}_{endo}$ for nonrecursive models and is obtained by using ${\hat{\boldsymbol{\Phi }}}_{exo}$, ${\hat{\mathbf{B}}}$, ${\hat{\boldsymbol{\Gamma }}}$, ${\hat{\boldsymbol{\Psi }}}$ in (18) for recursive ones.

**Step 5.** Using final estimators, the model-implied correlation matrix ${\hat{\mathbf{R}}} = \left\{ {\hat{\rho }_{jk} } \right\}$ is obtained from (24) or (28).

**Step 6.** The model-implied covariance matrix ${\hat{\boldsymbol{\Sigma }}} = \left\{ {{\hat{\mathbf{\Sigma }}}_{jk} } \right\}$ is computed by using

36 $$\begin{gathered} {\hat{\boldsymbol{\Sigma }}}_{jk} = \hat{\rho }_{jk} {\hat{\boldsymbol{\uplambda }}}_{j} {\hat{\boldsymbol{\uplambda }}}_{k}^{t} ,{\text{ for }}j \ne k,{\text{ and}} \hfill \\\\ {\hat{\boldsymbol{\Sigma }}}_{jj} = \left\{ {\begin{array}{*{20}c} {{\hat{\boldsymbol{\uplambda }}}_{j} {\hat{\boldsymbol{\uplambda }}}_{j}^{t} + {\hat{\boldsymbol{\Theta }}}_{j} ,} & {\text{for a reflective block,}} \\ {{\mathbf{S}}_{jj} {,}} & {{\text{for a formative block}}{.}} \\ \end{array} } \right. \hfill \\ \end{gathered}$$

### Test-of-fit, test of significance and improper solutions for SVD-SEM

A measure of model fit is obtained by comparing the covariance matrix ${\boldsymbol{S}}$ of the whole set of indicators to the model-implied covariance matrix ${\hat{\boldsymbol{\Sigma }}}$. The Euclidean distance $d\left( {{\hat{\boldsymbol{\Sigma }}},{\mathbf{S}}} \right) = \sqrt {\frac{1}{2}{\mathrm{trace}}\left[ {\left( {{\mathbf{S}} - {\hat{\boldsymbol{\Sigma }}}} \right)^{2} } \right]}$ is considered. Following the Bollen-Stine bootstrapping method (Bollen and Stine 1993), used in the framework of PLSc in Dijkstra and Henseler (2015), this distance can be used for validating the structural equation model with factors and composites.

The test of hypothesis $H_{0} :g( {{\boldsymbol{\uptheta}}}) = 0$ can be carried out for any smooth function *g*. A simple based-resampling procedure is proposed. *B* bootstrap samples are drawn from the original sample with the belonging condition that the SVD-SEM output of each bootstrap sample does not contain any improper solutions. A sample of *B* values of $g( {{\hat{\boldsymbol{\theta }}}})$ is obtained and the standard deviation of this sample is derived. The *p*-value is computed by assuming that the ratio of $g( {{\hat{\boldsymbol{\theta }}}})$ to this standard deviation follows the standard normal distribution.

Improper solutions correspond to situations where parameter estimates at the sample level do not have the properties of the population parameters. When the data satisfy the structural equation model, consistency of the estimators implies that the probability of occurrence of improper solutions tends to zero for large samples. We observe this behavior in the Monte Carlo simulation discussed in Sect. 6 (see Table 9).

## Maximum likelihood estimation in the framework of the basic design

We study in this section maximum-likelihood estimator of the basic design. For models containing factors only, standard ML-SEM can be used. We consider a situation where the model contains factors and composites. The model-implied covariance matrix ${{\boldsymbol{\Sigma}}}\left( {{\boldsymbol{\uptheta}}} \right)$ is given by Eq. (30). In this equation, for each formative block, the constraint ${{\boldsymbol{\uplambda}}}_{j}^{t} {{\boldsymbol{\Sigma}}}_{jj}^{ - 1} {{\boldsymbol{\uplambda}}}_{j} = 1$ is satisfied and ${{\boldsymbol{\Theta}}}_{j}$ is set to **0**. Let *t* be the number of parameters in ${{\boldsymbol{\uptheta}}}$. Assuming now that the vector ${\boldsymbol{y}}$ of observed variables has a multinormal distribution, we consider the maximum-likelihood fitting function

37 $$F\left( {{\boldsymbol{\uptheta}}} \right) = \log \left| {{{\boldsymbol{\Sigma}}}\left( {{\boldsymbol{\uptheta}}} \right)} \right| + {\mathrm{tr}}\left( {{{\boldsymbol{\Sigma}}}\left( {{\boldsymbol{\uptheta}}} \right)^{ - 1} {\mathbf{S}}} \right) - \log \left| {\mathbf{S}} \right| - p,$$

where ${\mathbf{S}}$ is the usual unbiased sample covariance matrix. Assume that *r* blocks are formative. The *r* constraints ${{\boldsymbol{\uplambda}}}_{j}^{t} {{\boldsymbol{\Sigma}}}_{jj}^{ - 1} {{\boldsymbol{\uplambda}}}_{j} = 1$ are expressed as *r* independent restrictions on ${{\boldsymbol{\uptheta}}}$: $h_{1} \left( {{\boldsymbol{\uptheta}}} \right) = h_{2} \left( {{\boldsymbol{\uptheta}}} \right) = ... = h_{r} \left( {{\boldsymbol{\uptheta}}} \right) = 0$. Considering these restrictions, we assume that $t - r$ free parameters can be extracted from ${{\boldsymbol{\uptheta}}}$. The restricted maximum-likelihood estimate ${\hat{\boldsymbol{\theta }}}_{{{\mathrm{RML}}}}$ is obtained by minimizing the fitting function $F\left( {{\boldsymbol{\uptheta}}} \right)$ subject to the constraints $h_{1} \left( {{\boldsymbol{\uptheta}}} \right) = h_{2} \left( {{\boldsymbol{\uptheta}}} \right) = ... = h_{r} \left( {{\boldsymbol{\uptheta}}} \right) = 0$. The properties established for the method of maximum likelihood (consistency, asymptotic unbiasedness, asymptotic efficiency, and asymptotic normal distribution) hold for restricted ML estimates (Silvey 1975).

The information matrix for a single observation of a multinormal distribution is given by the formula

38 $${\mathbf{I}}_{{{\boldsymbol{\uptheta}}}} = \left\{ {\frac{1}{2}{\mathrm{tr}} \left[ {{{\boldsymbol{\Sigma}}}\left( {{\boldsymbol{\uptheta}}} \right)^{ - 1} \frac{{\partial {{\boldsymbol{\Sigma}}}\left( {{\boldsymbol{\uptheta}}} \right)}}{{\partial \theta_{i} }}{{\boldsymbol{\Sigma}}}\left( {{\boldsymbol{\uptheta}}} \right)^{ - 1} \frac{{\partial {{\boldsymbol{\Sigma}}}\left( {{\boldsymbol{\uptheta}}} \right)}}{{\partial \theta_{j} }}} \right]} \right\}.$$

We denote by ${\mathbf{H}}_{{{\boldsymbol{\uptheta}}}}$ the $t \times r$ matrix of partial derivatives $\partial h_{j} \left( {{\boldsymbol{\uptheta}}} \right)/\partial \theta_{i}$. We assume that the matrix ${\mathbf{I}}_{{{\boldsymbol{\uptheta}}}} + {\mathbf{H}}_{{{\boldsymbol{\uptheta}}}} {\mathbf{H}}_{{{\boldsymbol{\uptheta}}}}^{t}$ is non-singular. Then, the asymptotic distribution of the restricted maximum-likelihood estimator ${\hat{\boldsymbol{\theta }}}_{{{\mathrm{RML}}}}$ of ${{\boldsymbol{\uptheta}}}$ is given in Silvey (1975, p. 82):

39 $$\sqrt N \left( {{\hat{\boldsymbol{\theta }}}_{{{\mathrm{RML}}}} - {{\boldsymbol{\uptheta}}}} \right) \xrightarrow{D} N\left( {{\mathbf{0}},{\mathbf{P}}_{{{\boldsymbol{\uptheta}}}} } \right),$$

where *N* is the sample size and ${\mathbf{P}}_{{{\boldsymbol{\uptheta}}}}$ the leading $t \times t$ submatrix in $$\left[ {\begin{array}{*{20}c} {{\mathbf{I}}_{{{\boldsymbol{\uptheta}}}} + {\mathbf{H}}_{{{\boldsymbol{\uptheta}}}} {\mathbf{H}}_{{{\boldsymbol{\uptheta}}}}^{t} } & {{\mathbf{H}}_{{{\boldsymbol{\uptheta}}}} } \\ {{\mathbf{H}}_{{{\boldsymbol{\uptheta}}}}^{t} } & {\mathbf{0}} \\ \end{array} } \right]^{ - 1}$$.

Under the assumption that the distribution of ${\boldsymbol{y}}$ is multinormal, the likelihood-ratio test can be used to test the validity of the model studied in this paper. Minus twice the logarithm of the likelihood-ratio is equal to $\left( {N - 1} \right)F\left( {{\hat{\boldsymbol{\theta }}}_{{{\mathrm{RML}}}} } \right)$. Therefore, assuming the model true, the asymptotic distribution of $\left( {N - 1} \right)F\left( {{\hat{\boldsymbol{\theta }}}_{{{\mathrm{RML}}}} } \right)$ is a chi-square with $\frac{1}{2}p\left( {p + 1} \right) - t + r$ degrees of freedom. We also consider the hypothesis $H_{0} :g_{1} \left( {{\boldsymbol{\uptheta}}} \right) = ... = g_{k} \left( {{\boldsymbol{\uptheta}}} \right) = 0$ for any set of independent smooth functions $g_{1} ,...,g_{k}$. Let ${\hat{\boldsymbol{\theta }}}_{{{\mathrm{RML}}}} \left( {H_{0} } \right)$ be the restricted ML estimate of ${{\boldsymbol{\uptheta}}}$ under $H_{0}$. Then, assuming $H_{0}$ true, the asymptotic distribution of $\left( {N - 1} \right)\left( {F\left( {{\hat{\boldsymbol{\theta }}}_{{{\mathrm{RML}}}} \left( {H_{0} } \right)} \right) - F\left( {{\hat{\boldsymbol{\theta }}}_{{{\mathrm{RML}}}} } \right)} \right)$ is a chi-square with $k$ degrees of freedom. The asymptotic distribution of ${\hat{\boldsymbol{\theta }}}_{{{\mathrm{RML}}}} \left( {H_{0} } \right)$ is also provided in Silvey (p.82). Let ${\mathbf{G}}_{{{\boldsymbol{\uptheta}}}}$ be the $t \times k$ matrix of partial derivatives $\partial g_{j} \left( {{\boldsymbol{\uptheta}}} \right)/\partial \theta_{i}$. Then, $\sqrt N \left( {{\hat{\boldsymbol{\theta }}}_{{{\mathrm{RML}}}} \left( {H_{0} } \right) - {{\boldsymbol{\uptheta}}}} \right) \xrightarrow D N\left( {{\mathbf{0}},{\mathbf{P}}_{{{\boldsymbol{\uptheta}}}} } \right)$, where ${\mathbf{P}}_{{{\boldsymbol{\uptheta}}}}$ is now the leading $t \times t$ submatrix in $$\left[ {\begin{array}{*{20}c} {{\mathbf{I}}_{{{\boldsymbol{\uptheta}}}} + {\mathbf{H}}_{{{\boldsymbol{\uptheta}}}} {\mathbf{H}}_{{{\boldsymbol{\uptheta}}}}^{t} } & {\left[ {{\mathbf{H}}_{{{\boldsymbol{\uptheta}}}} ,{\mathbf{G}}_{{{\boldsymbol{\uptheta}}}} } \right]} \\ {\left[ {{\mathbf{H}}_{{{\boldsymbol{\uptheta}}}} ,{\mathbf{G}}_{{{\boldsymbol{\uptheta}}}} } \right]^{t} } & {\mathbf{0}} \\ \end{array} } \right]^{ - 1}$$.

## Single block regression predictor of a factor

The block component $\hat{\xi }_{j} = {{\boldsymbol{\uplambda}}}_{j}^{t} {{\boldsymbol{\Sigma}}}_{jj}^{ - 1} {\boldsymbol{y}}_{j}$ is the single-block regression predictor of factor $\xi_{j}$ based on ${\boldsymbol{y}}_{j}$ (linear minimum mean square error (MMSE) predictor). The resulting reliability coefficient of $\hat{\xi }_{j}$ is $\rho_{{\hat{\xi }_{j} \hat{\xi }_{j} }} = cor^{2} \left( {\hat{\xi }_{j} ,\xi_{j} } \right)$ which is equal to ${{\boldsymbol{\uplambda}}}_{j}^{t} {{\boldsymbol{\Sigma}}}_{jj}^{ - 1} {{\boldsymbol{\uplambda}}}_{j}^{{}}$. The regression predictor $\hat{\xi }_{j}$ is also a block component with the largest possible reliability coefficient.

Using the Sherman-Morrison formula, we obtain more explicit formulas: $\hat{\xi }_{j} = {{\boldsymbol{\uplambda}}}_{j}^{t} {{\boldsymbol{\Theta}}}_{j}^{ - 1} {\boldsymbol{y}}_{j} /\left( {1 + {{\boldsymbol{\uplambda}}}_{j}^{t} {{\boldsymbol{\Theta}}}_{j}^{ - 1} {{\boldsymbol{\uplambda}}}_{j} } \right)$ and $\rho_{{\hat{\xi }_{j} \hat{\xi }_{j} }} = {{\boldsymbol{\uplambda}}}_{j}^{t} {{\boldsymbol{\Theta}}}_{j}^{ - 1} {{\boldsymbol{\uplambda}}}_{j} /\left( {1 + {{\boldsymbol{\uplambda}}}_{j}^{t} {{\boldsymbol{\Theta}}}_{j}^{ - 1} {{\boldsymbol{\uplambda}}}_{j} } \right)$. As factors have unit variance in this paper, the standardized loading $\lambda_{jh}^{s} = \lambda_{jh} /\sqrt {var\left( {y_{jh} } \right)} = cor\left( {y_{jh} ,\xi_{j} } \right)$ is also the standardized validity coefficient and its square the reliability coefficient of $y_{jh}$ (Bollen 1989). It is instructive to write $\hat{\xi }_{j}$ and $\rho_{{\hat{\xi }_{j} \hat{\xi }_{j} }}$ in terms of standardized loadings and standardized indicators $y_{jh}^{s} = y_{jh} /\sqrt {var\left( {y_{jh} } \right)}$. Setting $\upsilon_{j} = \sum\limits_{h} {\lambda_{jh}^{s2} /\left( {1 - \lambda_{jh}^{s2} } \right)}$, we obtain $\hat{\xi }_{j} = \frac{1}{{1 + \upsilon_{j} }}\sum\limits_{h} {\left( {\lambda_{jh}^{s} /\left( {1 - \lambda_{jh}^{s2} } \right)} \right)y_{jh}^{s} }$. The reliability of $\hat{\xi }_{j}$ boils down to $\rho_{{\hat{\xi }_{j} \hat{\xi }_{j} }} = \frac{{\upsilon_{j} }}{{1 + \upsilon_{j} }}$ and is an increasing function of the reliability coefficients of the block indicators. Assuming that $\ell_{j} \mathop { = \min }\limits_{ \, h} \lambda_{jh}^{s2}> 0$, $\rho_{{\hat{\xi }_{j} \hat{\xi }_{j} }} \ge \frac{{p_{j} \ell_{j} }}{{1 - \ell_{j} + p_{j} \ell_{j} }}$. Therefore, assuming a positive lower bound for all $\lambda_{jh}^{s2} ,$ $\rho_{{\hat{\xi }_{j} \hat{\xi }_{j} }} = cor^{2} \left( {\hat{\xi }_{j} ,\xi_{j} } \right)$ tends to 1 as the number $p_{j}$ of indicators in block *j* tends to infinity. This is what Herman Wold calls “consistency at large”.

## Monte-Carlo simulation

To assess the quality of the estimators provided by SVD-SEM and RML-SEM a Monte-Carlo simulation was conducted on a model proposed by Dijkstra and Henseler (2015). In that paper, all LVs are factors. In this simulation, we modify the nature of the LVs by introducing factors and composites.

### Setup

**FIGURE 2:**
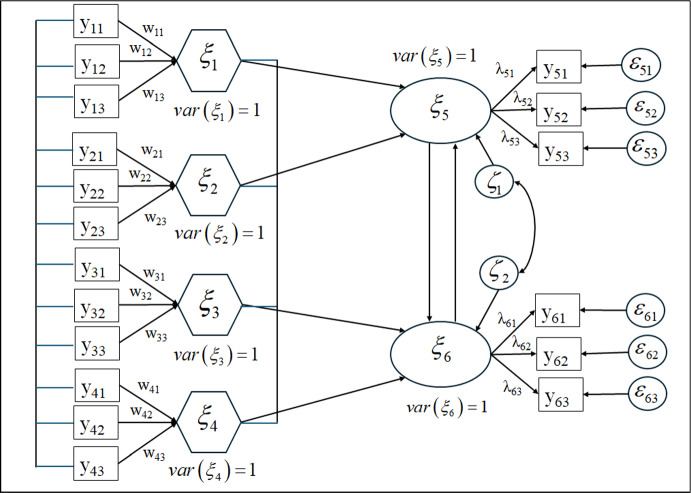
Path diagram associated with Model (40) for factors and composites. Constraints of the basic design are assumed

Endogenous and exogenous LVs are linked to each other through the following structural equation:

40 $$\left[ {\begin{array}{*{20}c} 1 & { - 0.25} \\ { - 0.50} & 1 \\ \end{array} } \right]\left[ {\begin{array}{*{20}c} {\xi_{5} } \\ {\xi_{6} } \\ \end{array} } \right] = \left[ {\begin{array}{*{20}c} { - 0.30} & {0.50} & 0 & 0 \\ 0 & 0 & {0.50} & {0.25} \\ \end{array} } \right]\left[ {\begin{array}{*{20}c} {\xi_{1} } \\ {\xi_{2} } \\ {\xi_{3} } \\ {\xi_{4} } \\ \end{array} } \right] + \left[ {\begin{array}{*{20}c} {\zeta_{1} } \\ {\zeta_{2} } \\ \end{array} } \right],$$

where all variables $\xi_{j}$ have zero mean and unit variance. According to Wold (1985, Arrow scheme IV*, p. 582, and comments on Modes A and B used in the PLS algorithm, p. 585), it is often appropriate to choose Mode A for endogenous blocks and Mode B for exogenous ones. In the framework of this paper, this comes down to consider endogenous blocks as reflective and exogenous blocks as formative. We follow these recommendations for Model (40) depicted in Fig. 2 where each block contains three indicators. The formative blocks are represented by hexagons. The correlation matrices of the exogenous variables and of the endogenous ones are set to:

$${{\boldsymbol{\Phi}}}_{exo} = \left[ {\begin{array}{*{20}c} 1 & {} & {} & {} \\ {0.50} & 1 & {} & {} \\ {0.50} & {0.50} & 1 & {} \\ {0.50} & {0.50} & {0.50} & 1 \\ \end{array} } \right]\quad {\mathrm{and}}\quad {{\boldsymbol{\Phi}}}_{endo} = \left[ {\begin{array}{*{20}c} 1 & {} \\ {\sqrt {0.50} } & 1 \\ \end{array} } \right].$$

Then, using (24) and (23), the model-implied correlation matrix and the covariance matrix of the disturbance vector are given by

$$\begin{aligned} {\mathbf{R}} & = \begin{array}{*{20}c} {} \\ \end{array} \left[ {\begin{array}{*{20}c} 1 & {} & {} & {} & {} & {} \\ {0.5} & 1 & {} & {} & {} & {} \\ {0.5} & {0.5} & 1 & {} & {} & {} \\ {0.5} & {0.5} & {0.5} & 1 & {} & {} \\ {0.0500} & {0.5071} & {0.2929} & {0.2571} & 1 & {} \\ {0.4000} & {0.6286} & {0.7714} & {0.6286} & {\sqrt {.5} } & 1 \\ \end{array} } \right]\\\\ & {\mathrm{and}} \quad\quad {{\boldsymbol{\Psi}}} = \left[ {\begin{array}{*{20}c} {0.5189} & {} \\ { - 0.0295} & {0.1054} \\ \end{array} } \right]. \\ \end{aligned}$$

Using (29), we obtain $R_{1}^{2} = 0.4811$ and $R_{2}^{2} = 0.8946$.

We now describe the measurement model in the framework of the basic design. Each composite $\xi_{j}$ is built up by three indicators ${\boldsymbol{y}}_{j} = \left( {y_{j1} ;y_{j2} ;y_{j3} } \right)$ with $${{\boldsymbol{\Sigma}}}_{jj} = \left[ {\begin{array}{*{20}c} 1 & {} & {} \\ {0.30} & 1 & {} \\ {0.40} & {0.50} & 1 \\ \end{array} } \right]$$, $j = 1,...,4$. The weight vectors are set to ${\mathbf{w}}_{1} { = }{\mathbf{w}}_{2} \propto \left( {1 \, ; \, 1 \, ; \, 1} \right)$ and ${\mathbf{w}}_{3} { = }{\mathbf{w}}_{4} \propto \left( {1 \, ; \, 2 \, ; \, 3} \right)$; imposing the constraint ${\mathbf{w}}_{j}^{t} {{\boldsymbol{\Sigma}}}_{jj} {\mathbf{w}}_{j} = 1$, this yields ${\mathbf{w}}_{1} = {\mathbf{w}}_{2} = \left( {0.4303 \, ; \, 0.4303 \, ; \, 0.4303} \right)$ and ${\mathbf{w}}_{3} = {\mathbf{w}}_{4} = \left( {0.2058 \, ; \, 0.4117 \, ; \, 0.6175} \right)$. The loading vectors for composites are deduced from (6): ${{\boldsymbol{\uplambda}}}_{1} = {{\boldsymbol{\uplambda}}}_{2} = \left( {0.7316 \, ; \, 0.7746 \, ; \, 0.8176} \right)$ and ${{\boldsymbol{\uplambda}}}_{3} = {{\boldsymbol{\uplambda}}}_{4} = \left( {0.5763 \, ; \, 0.7822 \, ; \, 0.9057} \right)$. The factors $\xi_{5} ,\xi_{6}$ are measured indirectly by three zero-mean and unit-variance indicators ${\boldsymbol{y}}_{j} = \left( {y_{j1} ;y_{j2} ;y_{j3} } \right)$ with all loadings set to 0.7. All measurement errors are mutually uncorrelated and uncorrelated with all unobserved variables and composite indicators. All this leads to

$${{\boldsymbol{\Sigma}}}_{55} = {{\boldsymbol{\Sigma}}}_{66} = \left[ {\begin{array}{*{20}c} 1 & {} & {} \\ {0.49} & 1 & {} \\ {0.49} & {0.49} & 1 \\ \end{array} } \right].$$

The covariance matrix ${{\boldsymbol{\Sigma}}} = \left\{ {{{\boldsymbol{\Sigma}}}_{jk} } \right\}$ of the whole set of 18 indicators can now be computed from (8) by setting ${{\boldsymbol{\Sigma}}}_{jk} = \rho_{jk} {{\boldsymbol{\uplambda}}}_{j} {{\boldsymbol{\uplambda}}}_{k}^{t}$ for $j \ne k$. Then, we generate 1000 samples of $N = 300$ observations by random drawings from $N\left( {\mathbf{0},{{\boldsymbol{\Sigma}}}} \right)$.

### Results

SVD-SEM and RML-SEM are used on each sample to estimate model parameters. For RML-SEM, we use the derivative-free solver for constrained nonlinear optimization SOLNP proposed by Ye (1987, 1989) and available in R [R Core Team 2024] using the *solnp* function of the *Rsolnp* package (Ghalanos and Theussl 2015). We initialize the SOLNP algorithm with ${\mathbf{S}}_{jj}$ for formative blocks and with the output of SVD-SEM for the other parameters in ${{\boldsymbol{\uptheta}}}$. At convergence of the SOLNP algorithm, the restricted ML estimate ${\hat{\boldsymbol{\theta }}}_{{{\mathrm{RML}}}}$ is obtained. Then, using ${\hat{\boldsymbol{\theta }}}_{{{\mathrm{RML}}}}$ and the *jacobian* function of the *numeDeriv* R package (Gilbert and Varadhan 2019), we can compute numerical approximations of ${\mathbf{I}}_{{{\boldsymbol{\uptheta}}}}$ and ${\mathbf{H}}_{{{\boldsymbol{\uptheta}}}}$ from which we deduce an estimate ${\hat{\mathbf{P}}}_{{{\boldsymbol{\uptheta}}}}$ of ${\mathbf{P}}_{{{\boldsymbol{\uptheta}}}}$.

In Table 1, we observe that the restricted ML estimates ${\hat{\boldsymbol{\Sigma }}}_{11} ,...,{\hat{\boldsymbol{\Sigma }}}_{44}$ of ${{\boldsymbol{\Sigma}}}_{11} ,...,{{\boldsymbol{\Sigma}}}_{44}$ are very close to the usual unbiased estimates ${\mathbf{S}}_{11} ,...,{\mathbf{S}}_{44}$. This suggests that imposing the constraints ${\hat{\boldsymbol{\Sigma }}}_{jj} = \mathbf{S}_{jj}$ for formative blocks in RML-SEM can be considered. The SEMFC R package and the code used to produce the results are freely available on GitHub (https://github.com/Tenenhaus/SEMFC).

**Table 1:** Estimate of covariance matrices for formative blocks

	SVD-SEM	RML-SEM	
	$d\left( {{\mathbf{S}}_{jj} ,{{\boldsymbol{\Sigma}}}_{jj} } \right)$	Mean $d\left( {{\hat{\boldsymbol{\Sigma }}}_{jj} ,{{\boldsymbol{\Sigma}}}_{jj} } \right)$	Mean $d\left( {{\mathbf{S}}_{jj} ,{\hat{\boldsymbol{\Sigma }}}_{{jj}}} \right)$
${{\boldsymbol{\Sigma}}}_{11}$	0.1377	0.1377	0.000029
${{\boldsymbol{\Sigma}}}_{22}$	0.1388	0.1388	0.000026
${{\boldsymbol{\Sigma}}}_{33}$	0.1423	0.1423	0.000029
${{\boldsymbol{\Sigma}}}_{44}$	0.1375	0.1375	0.000028

We provide detailed comments on Table 2. The comments for Tables 3, 4, 5 and 6 are quite similar. The loading estimators $\hat{\lambda }_{ij}^{{{\mathrm{SVD}}}}$ and $\hat{\lambda }_{ij}^{{{\mathrm{RML}}}}$ are consistent, asymptotically unbiased, asymptotically normally distributed and for the latter asymptotically efficient. For each sample $\ell=1, \ldots, 1000$, estimates $\hat{\lambda }_{ij}^{{{\mathrm{SVD}}}}\left(\ell\right)$, $\hat{\lambda }_{ij}^{{{\mathrm{RML}}}} \left( \ell \right)$ and $\hat{\mathbf{P}}_{\boldsymbol{\uptheta}}\left(\ell \right)$ are obtained.

**Table 2:** Loading estimates

Parameter	Population value	Mean of $\hat{\lambda }_{ij}^{{}} \left( \ell \right)$, $\ell = 1,...,1000$		Standard Deviation of $\hat{\lambda }_{ij}^{{}} \left( \ell \right)$, $\ell = 1,...,1000$		Mean of $\hat{{{\sigma}}}\left( {\hat{\lambda }_{ij}^{{{\mathrm{RML}}}} } \right)\left[ \ell \right]$, $\ell = 1,...,1000$
		(1) SVD-SEM	(2) RML-SEM	(3) SVD-SEM	(4) RML-SEM	(5) RML-SEM
$\lambda_{11}$	0.7316	0.726	0.728	0.075	0.069	0.068
$\lambda_{12}$	0.7746	0.771	0.771	0.071	0.066	0.064
$\lambda_{13}$	0.8176	0.808	0.810	0.064	0.059	0.061
$\lambda_{21}$	0.7316	0.728	0.728	0.066	0.066	0.063
$\lambda_{22}$	0.7746	0.774	0.774	0.063	0.063	0.061
$\lambda_{23}$	0.8176	0.815	0.814	0.061	0.060	0.058
$\lambda_{31}$	0.5763	0.574	0.574	0.073	0.071	0.068
$\lambda_{32}$	0.7822	0.779	0.779	0.061	0.058	0.058
$\lambda_{33}$	0.9057	0.901	0.902	0.052	0.051	0.050
$\lambda_{41}$	0.5764	0.575	0.574	0.080	0.078	0.075
$\lambda_{42}$	0.7822	0.777	0.777	0.066	0.064	0.063
$\lambda_{43}$	0.9057	0.899	0.900	0.054	0.053	0.053
$\lambda_{51}$	0.7	0.699	0.700	0.071	0.057	0.057
$\lambda_{52}$	0.7	0.694	0.695	0.071	0.058	0.057
$\lambda_{53}$	0.7	0.699	0.700	0.069	0.056	0.057
$\lambda_{61}$	0.7	0.697	0.698	0.056	0.054	0.053
$\lambda_{62}$	0.7	0.700	0.700	0.058	0.056	0.053
$\lambda_{63}$	0.7	0.699	0.699	0.055	0.055	0.053
MEAN				0.065	0.061	0.060

**Table 3:** Estimate of measurement error variances

Parameter	Population value	Mean of estimate		Standard deviation of estimate		Mean of standard error of estimate
		SVD-SEM	RML-SEM	SVD-SEM	RML-SEM	RML-SEM
$\theta_{51}$	0.51	0.505	0.506	0.078	0.058	0.056
$\theta_{52}$	0.51	0.511	0.510	0.081	0.056	0.056
$\theta_{53}$	0.51	0.508	0.509	0.081	0.056	0.056
$\theta_{61}$	0.51	0.510	0.508	0.053	0.050	0.048
$\theta_{62}$	0.51	0.506	0.507	0.050	0.049	0.048
$\theta_{63}$	0.51	0.506	0.507	0.050	0.047	0.048
MEAN				0.066	0.053	0.052

**Table 4:** Weight estimates for formative blocks

Parameter	Population value	Mean of estimate		Standard deviation of estimate	
		SVD-SEM	RML-SEM	SVD-SEM	RML-SEM
$w_{11}$	0.4303	0.426	0.427	0.084	0.072
$w_{12}$	0.4303	0.433	0.432	0.087	0.076
$w_{13}$	0.4303	0.428	0.430	0.092	0.079
$w_{21}$	0.4303	0.428	0.429	0.066	0.065
$w_{22}$	0.4303	0.430	0.431	0.072	0.071
$w_{23}$	0.4303	0.429	0.428	0.075	0.074
$w_{31}$	0.2058	0.207	0.207	0.065	0.059
$w_{32}$	0.4117	0.413	0.413	0.065	0.059
$w_{33}$	0.6175	0.615	0.616	0.066	0.060
$w_{41}$	0.2058	0.211	0.208	0.082	0.078
$w_{42}$	0.4117	0.410	0.410	0.078	0.075
$w_{43}$	0.6175	0.614	0.616	0.079	0.077
MEAN				0.076	0.070

**Table 5:** Estimate of structural equations

Parameter	Population value	Mean of estimate		Standard deviation of estimate		Mean of standard error of estimate
		SVD-SEM	RML-SEM	SVD-SEM	RML-SEM	RML-SEM
$\gamma_{11}$	− 0.3000	− 0.302	− 0.305	0.065	0.062	0.060
$\gamma_{12}$	0.5000	0.501	0.505	0.078	0.078	0.078
$\gamma_{23}$	0.5000	0.500	0.501	0.053	0.050	0.050
$\gamma_{24}$	0.2500	0.249	0.249	0.054	0.049	0.047
$\beta_{12}$	0.2500	0.251	0.246	0.096	0.094	0.093
$\beta_{21}$	0.5000	0.502	0.503	0.086	0.089	0.087
MEAN				0.072	0.070	0.069
$\psi_{11}$	0.5189	0.515	0.516	0.077	0.077	NA
$\psi_{22}$	0.1054	0.107	0.107	0.048	0.048	NA
$\psi_{12}$	− 0.0295	− 0.031	− 0.030	0.072	0.074	NA
$R_{1}^{2}$	0.4811	0.485	0.484	0.077	0.077	NA
$R_{2}^{2}$	0.8946	0.893	0.893	0.048	0.048	NA

**Table 6:** Estimate of factor/composite correlations

Parameter	Population value	Mean of estimate		Standard deviation of estimate		Mean of standard error of estimate
		SVD-SEM	RML-SEM	SVD-SEM	RML-SEM	RML-SEM
$\rho_{12}$	0.5000	0.504	0.503	0.043	0.043	0.043
$\rho_{13}$	0.5000	0.501	0.500	0.044	0.044	0.043
$\rho_{14}$	0.5000	0.502	0.502	0.043	0.043	0.043
$\rho_{15}$	0.0500	0.050	0.047	0.072	0.069	NA
$\rho_{16}$	0.4000	0.401	0.400	0.054	0.054	NA
$\rho_{23}$	0.5000	0.502	0.501	0.043	0.043	0.043
$\rho_{24}$	0.5000	0.503	0.503	0.043	0.044	0.043
$\rho_{25}$	0.5071	0.506	0.507	0.054	0.053	NA
$\rho_{26}$	0.6286	0.630	0.630	0.043	0.043	NA
$\rho_{34}$	0.5000	0.501	0.500	0.044	0.043	0.043
$\rho_{35}$	0.2929	0.294	0.291	0.060	0.061	NA
$\rho_{36}$	0.7714	0.772	0.772	0.036	0.035	NA
$\rho_{45}$	0.2571	0.259	0.256	0.052	0.052	NA
$\rho_{46}$	0.6286	0.629	0.628	0.047	0.045	NA
$\rho_{56}$	0.7071	0.706	0.706	0.050	0.051	0.052
MEAN				0.049	0.048	
MEAN in $\boldsymbol{\Phi}_{endo}$ and $\boldsymbol{\Phi}_{exo}$				0.044	0.044	0.044

Columns (1) and (2) report the means of the estimates $\hat{\lambda }_{ij}^{{{\mathrm{SVD}}}}\left(\ell \right)$ and $\hat{\lambda }_{ij}^{{{\mathrm{RML}}}} \left( \ell \right)$ and suggest that SVD-SEM and RML-SEM produce virtually unbiased estimates of the loadings.

Columns (3) and (4) report estimates of the standard errors ${{{\sigma}}} \left( {\hat{\lambda}_{ij}^{{{\mathrm{SVD}}}}} \right)$ and ${{{\sigma}}}\left( {\hat{\lambda }_{ij}^{{{\mathrm{RML}}}} } \right)$, computed as the standard deviations of the estimates $\hat{\lambda }_{ij}^{{{\mathrm{SVD}}}}\left(\ell \right)$ and $\hat{\lambda }_{ij}^{{{\mathrm{RML}}}} \left( \ell \right)$. As the sample means are very close to the value of the parameters at the population level, these standard deviations measure the quality of the estimates. As expected (efficiency of maximum likelihood estimators), RML-SEM produces slightly more precise results than SVD-SEM.

Furthermore, for each sample $\ell = 1, \ldots, 1000$, an estimate $\hat{{{\sigma}}}\left( {\hat{\lambda }_{ij}^{{{\mathrm{RML}}}} } \right)\left[ \ell \right]$ of $\sigma \left( {\hat{\lambda }_{ij}^{\mathrm{RML}} } \right)$ can be computed from (39) as the corresponding element of $\left[ {diag\left( {{\hat{\mathbf{P}}}_{{{\boldsymbol{\uptheta}}}} \left( \ell \right)} \right)/300} \right]^{1/2}$. The means on the 1000 samples of these $\hat{{{\sigma}}}\left( {\hat{\lambda }_{ij}^{{{\mathrm{RML}}}} } \right)\left[ \ell \right]$, reported in column (5), are slightly smaller than the previous estimates given in column (4), except in two cases.

The overall test-of-fit is carried out as follows. For SVD-SEM, we applied the Bollen-Stine bootstrapping method for *N* = 300, 600, 1200 observations. For each size *N*, 1000 normal samples are generated by random drawings from $N\left( {\mathbf{0},{{\boldsymbol{\Sigma}}}} \right)$. For each of these samples, the Bollen-Stine bootstrap test procedure is carried out with 1000 bootstrap samples at the nominal level $\alpha = .05$. For RML-SEM, the parameter-vector ${{\boldsymbol{\uptheta}}}$ contains $t = 61$ elements. To ensure identifiability, the $r = 4$ constraints $h_{j} \left( {{\boldsymbol{\uptheta}}} \right) = {{\boldsymbol{\uplambda}}}_{j}^{t} {{\boldsymbol{\Sigma}}}_{jj}^{ - 1} {{\boldsymbol{\uplambda}}}_{j} - 1 = 0$, $j = 1,...,4$, are imposed. The number of degrees of freedom for the Chi-square test is $18 \times 19/2 - 61 + 4 = 114$. Results are given in Table 7. For *N* = 300 and 600, tests of fit for SVD-SEM are more cautious than desired: the empirical rejection probabilities are too small. We also observe that RML-SEM displays the well-known tendency to be too skeptical for small samples (see Hu et al., 1992). The same results have already been observed for PLSc in Dijkstra and Henseler (2015, Table 7). It helps to increase the sample size. For *N* = 1200, the empirical rejection probability is almost right for SVD-SEM. Table 7 also illustrates the convergence in probability of the empirical rejection probability to the nominal one.

**Table 7:** Empirical rejection probabilities using $d\left({{\hat {{\boldsymbol{\Sigma}}}},{\mathbf{S}}} \right)$ for SVD-SEM and Chi-square for RML-SEM. Percent of false alarms at the nominal level α = 5%

	Observations		
	300	600	1200
SVD-SEM	3.6	4.0	5.1
RML-SEM	7.6	5.8	4.3

Now we consider the test of hypothesis $H_{0} :\gamma_{12} = \gamma_{23}$. The 1000 samples used in this Monte-Carlo simulation come from a population with $\gamma_{12} = \gamma_{23} = .50$. and therefore, the hypothesis $H_{0}$ is true at population level.

*For SVD-SEM*. For each sample, $\hat{\gamma }_{12}^{{{\mathrm{SVD}}}} \left( \ell \right) - \hat{\gamma }_{23}^{{{\mathrm{SVD}}}} \left( \ell \right)$ is computed and the standard deviation $s\left( {\hat{\gamma }_{12}^{{{\mathrm{SVD}}}} - \hat{\gamma }_{23}^{{{\mathrm{SVD}}}} } \right)$ of these 1000 values is derived. Then, we compute for each sample $\ell$ the $\mathrm{p-value}\left( \ell \right) = P\left( {\left| z \right| \ge \frac{{\left| {\hat{\gamma }_{12}^{{{\mathrm{SVD}}}} \left( \ell \right) - \hat{\gamma }_{23}^{{{\mathrm{SVD}}}} \left( \ell \right)} \right|}}{{s\left( {\hat{\gamma }_{12}^{{{\mathrm{SVD}}}} - \hat{\gamma }_{23}^{{{\mathrm{SVD}}}} } \right)}}} \right)$, where $z \sim N\left( {0,1} \right)$.

*For RML-SEM*, two testing strategies can be used:

- Using the *z*-score: For each sample, the estimate of ${\mathrm{var}} \left( {\hat{\gamma }_{12}^{{{\mathrm{RML}}}} - \hat{\gamma }_{23}^{{{\mathrm{RML}}}} } \right)\left[ \ell \right] = {\mathrm{var}} \left( {\hat{\gamma }_{12}^{{{\mathrm{RML}}}} } \right)\left[ \ell \right] + {\mathrm{var}} \left( {\hat{\gamma }_{23}^{{{\mathrm{RML}}}} } \right)\left[ \ell \right] - 2{\mathrm{cov}} \left( {\hat{\gamma }_{12}^{{{\mathrm{RML}}}} ,\hat{\gamma }_{23}^{{{\mathrm{RML}}}} } \right)\left[ \ell \right]$ is deduced from (39) . Then, $\mathrm{p-value}\left( \ell \right) = P\left( {\left| z \right| \ge \frac{{\left| {\hat{\gamma }_{12}^{{{\mathrm{RML}}}} \left( \ell \right) - \hat{\gamma }_{23}^{{{\mathrm{RML}}}} \left( \ell \right)} \right|}}{{\sqrt {\widehat{{\mathrm{var}}}\left( {\hat{\gamma }_{12}^{{{\mathrm{RML}}}} - \hat{\gamma }_{23}^{{{\mathrm{RML}}}} } \right)\left[ \ell \right]} }}} \right)$.

- Using the LRT statistic: For each sample, the LRT statistic $\left( {N - 1} \right)\left( {F\left( {{\hat{\boldsymbol{\theta }}}_{{{\mathrm{RML}}}} \left( {H_{0} } \right)\left[ \ell \right]} \right) - F\left( {{\hat{\boldsymbol{\theta }}}_{{{\mathrm{RML}}}} \left[ \ell \right]} \right)} \right)$ is computed. Then, $\mathrm{p-value}\left( \ell \right) = P\left( {\chi_{1}^{2} \ge \left( {N - 1} \right)\left( {F\left( {{\hat{\boldsymbol{\theta }}}_{{{\mathrm{RML}}}} \left( {H_{0} } \right)\left[ \ell \right]} \right) - F\left( {{\hat{\boldsymbol{\theta }}}_{{{\mathrm{RML}}}} \left[ \ell \right]} \right)} \right)} \right)$.

We report in Table 8 the percent of false alarm at the nominal level $\alpha = 0.05$ for each hypothesis testing strategy. The results clearly show the validity of our approach. Results on improper solutions (Heywood cases) are given in Table 9. We note that SVD-SEM produces less improper solutions than RML-SEM, with a few exceptions. Due to consistency, the percentage of Heywood cases decreases as the sample size increases. In the same way, the percentage of nonconvergence for RML-SEM decreases as the sample size increases.

**Table 8:** Test of hypothesis $H_{0} :\gamma_{12} = \gamma_{23}$

	SVD-SEM	RML-SEM	
		z-score	LRT
Percent of false alarms at the nominal level α = 5%	4.9	5.0	4.7

**Table 9:** Results for Heywood cases computed on 1000 random samples. Percent of Heywood cases:

*N*	$\left| {\hat{\rho }_{ij} } \right|> 1$		$\hat{\theta }_{k\ell } < 0$		$R_{i}^{2} < 0$ or $R_{i}^{2}> 1$		*Percent of nonconvergence for RML-SEM*
	SVD-SEM	RML-SEM	SVD-SEM	RML-SEM	SVD-SEM	RML-SEM	
20	19.9	33.1	11.0	46.2	47.1	67.8	17.3
30	8.5	11.7	6.3	11.6	34.3	46.5	5.3
40	4.4	5.7	4.4	3.4	24.8	32.0	2.3
50	1.4	2.5	2.1	1.6	18.8	25.0	0.7
60	0.3	1.2	0.7	0.6	20.7	22.8	0.2
70	0.1	0.9	0.3	0.2	17.2	18.0	0.2
80	0	0.2	0.6	0	12.3	14.6	0
90	0	0.3	0.3	0.3	12.3	12.2	0
100	0	0.1	0.3	0	12.8	13.6	0
200	0	0	0.1	0	3.4	3.4	0
300	0	0	0	0	1.4	1.4	0
400	0	0	0	0	0.3	0.4	0
500	0	0	0	0	0.1	0	0
600	0	0	0	0	0	0	0

This study clearly confirms the feasibility of SVD-SEM in soft modeling. At the mathematical level, SVD-SEM outperforms PLS-SEM in all aspects. At the practical level, however, we have usually observed small differences between SVD-SEM and PLS-SEM. In this simulation, the results of PLS-SEM (not shown) and SVD-SEM are quite similar. Furthermore, within the framework of the basic design, the non-iterative approach SVD-SEM can be used to produce an initial solution for RML-SEM. When RML-SEM does not converge, SVD-SEM remains a good fallback solution.

## Conclusion

In this paper, we propose a unified framework for structural equation modeling with factors and composites for the basic design. The foundation of this unification lies in the fact that it is possible to express explicitly all model parameters in terms of elements of the covariance matrix of observed variables. This property facilitates the development of a new non-iterative algorithm called SVD-SEM. We show in this paper that SVD-SEM produces CAN estimators of the model parameters. SVD-SEM offers a sound statistically and computationally alternative to PLS-SEM (combination of PLSc and composite models). This approach allows a test-of-fit of the model and hypothesis testing of all model parameters by bootstrapping. Best single-block regression predictors of factors can also be obtained.

We also propose a maximum likelihood approach for the basic design. This leads to restricted maximum likelihood (RML-SEM) when the model contains some composites. We observed in a Monte Carlo simulation that SVD-SEM and RML-SEM produce very close results. SVD-SEM can be used as an initial solution for RML-SEM or alone when RML-SEM fails to converge. ML-SEM is difficult to use for large and complex models with many indicators and many latent variables. A comparison between SVD-SEM and ML-SEM in these situations would be instructive.

In high dimensional setting, sparse solutions may be desired to improve the interpretability of the results. Sparsity inducing penalties may be added to SVD-SEM or RML-SEM. Work in progress also includes extending SVD-SEM to categorical indicators.

## Data Availability

The SEMFC R package and the code used to produce the results are freely available on GitHub: https://github.com/Tenenhaus/SEMFC. All datasets used in this study are generated using simulated data.

## Appendix: CAN estimator of ${\mathbf{g}}\left( {{{\boldsymbol{\uplambda}}}_{1}^{*} ,...,{{\boldsymbol{\uplambda}}}_{J}^{*} ,{{\boldsymbol{\upsigma}}}} \right)$ for any smooth function g

Let $\, {\mathbf{s}} = {\mathrm{vech}}\left( {\mathbf{S}} \right)$ and $\, {{\boldsymbol{\upsigma}}} = {\mathrm{vech}}\left( {{\boldsymbol{\Sigma}}} \right)$ be the vectorized forms of, respectively, $\, {\mathbf{S}}$ and ${{\boldsymbol{\Sigma}}}$. Let ${{\boldsymbol{\uplambda}}}_{j}^{*} \left( {\mathbf{s}} \right)$ be the unit norm eigenvector of $\, {\mathbf{A}}_{j} \left( {\mathbf{s}} \right) = \sum\limits_{k = 1,k \ne j}^{J} {{\mathbf{S}}_{jk} {\mathbf{S}}_{kj} }$, with $\lambda_{j1}^{*} \left( {\mathbf{s}} \right)> 0$, corresponding to the largest eigenvalue. We first show that ${{\boldsymbol{\uplambda}}}_{j}^{*} \left( {\mathbf{s}} \right)$ is a CAN estimator of ${{\boldsymbol{\uplambda}}}_{j}^{*} = {{\boldsymbol{\uplambda}}}_{j}^{*} \left( {{\boldsymbol{\upsigma}}} \right)$. Let ${\mathbf{f}}_{j} \left( {{\mathbf{u}}_{j} ,{\mathbf{s}}} \right) = \frac{{{\mathbf{A}}_{j} \left( {\mathbf{s}} \right){\mathbf{u}}_{j} }}{{\left\| {{\mathbf{A}}_{j} \left( {\mathbf{s}} \right){\mathbf{u}}_{j} } \right\|}}$, where ${\mathbf{u}}_{j} \in {\mathbb{R}}^{{p_{j} }}$ with $u_{j1} \ge 0$, and ${\mathbf{F}}_{j} \left( {{\mathbf{u}}_{j} ,{\mathbf{s}}} \right) = {\mathbf{u}}_{j} - {\mathbf{f}}_{j} \left( {{\mathbf{u}}_{j} ,{\mathbf{s}}} \right)$. The eigenvector ${{\boldsymbol{\uplambda}}}_{j}^{*} \left( {\mathbf{s}} \right)$ of $\, {\mathbf{A}}_{j} \left( {\mathbf{s}} \right)$ satisfies the equation ${\mathbf{F}}_{j} \left( {{{\boldsymbol{\uplambda}}}_{j}^{*} \left( {\mathbf{s}} \right),{\mathbf{s}}} \right) = {\mathbf{0}}$. When ${\mathbf{s}}$ is sufficiently close to ${{\boldsymbol{\upsigma}}}$, the closest eigenvector of $\, {\mathbf{A}}_{j} \left( {\mathbf{s}} \right)$ to ${{\boldsymbol{\uplambda}}}_{j}^{*}$ is ${{\boldsymbol{\uplambda}}}_{j}^{*} \left( {\mathbf{s}} \right)$. This result and other useful results are detailed in the next propositions.

### Proposition 1

We assume ${{\boldsymbol{\Sigma}}}_{jk} = \rho_{jk} {{\boldsymbol{\uplambda}}}_{j} {{\boldsymbol{\uplambda}}}_{k}^{t}$ for $j \ne k$. We consider the function ${\mathbf{F}}_{j} \left( {{\mathbf{u}}_{j} ,{\mathbf{s}}} \right)$ in a neighborhood of $\left( {{{\boldsymbol{\uplambda}}}_{j}^{*} ,{{\boldsymbol{\upsigma}}}} \right)$. The function ${\mathbf{F}}_{j} \left( {{\mathbf{u}}_{j} ,{\mathbf{s}}} \right)$ satisfies ${\mathbf{F}}_{j} \left( {{{\boldsymbol{\uplambda}}}_{j}^{*} ,{{\boldsymbol{\upsigma}}}} \right) = {\mathbf{0}}$ and is continuously differentiable in the neighborhood of $\left( {{{\boldsymbol{\uplambda}}}_{j}^{*} ,{{\boldsymbol{\upsigma}}}} \right)$. We consider the continuous mapping ${{\boldsymbol{\uplambda}}}_{j}^{*} \left( {\mathbf{s}} \right)$. Then, there exist two spheres $U_{j} = \left\{ {{\mathbf{u}}_{j} |\left\| {{\mathbf{u}}_{j} - {{\boldsymbol{\uplambda}}}_{j}^{*} } \right\| < \delta_{j} } \right\}$ and $V_{j} = \left\{ {{\mathbf{s}}|\left\| {{\mathbf{s}} - {{\boldsymbol{\upsigma}}}} \right\| < \gamma_{j} } \right\}$ such that the following properties hold:

- (a) The equation ${\mathbf{F}}_{j} \left( {{\mathbf{u}}_{j} ,{\mathbf{s}}} \right) = {\mathbf{0}}$ is considered for $\left( {{\mathbf{u}}_{j} ,{\mathbf{s}}} \right) \in U_{j} \times V_{j}$. Then, for each ${\mathbf{s}} \in V_{j}$, the equation ${\mathbf{F}}_{j} \left( {{\mathbf{u}}_{j} ,{\mathbf{s}}} \right) = {\mathbf{0}}$ has ${{\boldsymbol{\uplambda}}}_{j}^{*} \left( {\mathbf{s}} \right) \in U_{j}$ for unique solution.
- (b) The mapping ${{\boldsymbol{\uplambda}}}_{j}^{*} \left( {\mathbf{s}} \right):V_{j} \to U_{j}$ is continuously differentiable and its derivative at ${\mathbf{s}} = {{\boldsymbol{\upsigma}}}$ is given by the formula:
  A1 $$\frac{{\partial {{\boldsymbol{\uplambda}}}_{j}^{*} \left( {\mathbf{s}} \right)}}{{\partial {\mathbf{s}}^{t} }}\left( {{\boldsymbol{\upsigma}}} \right) = \frac{{\partial {\mathbf{f}}_{j} }}{{\partial {\mathbf{s}}^{t} }}\left( {{{\boldsymbol{\uplambda}}}_{j}^{*} ,{{\boldsymbol{\upsigma}}}} \right).$$

**Proof of Point a:**

The implicit function theorem (see Copson 1968, Sect. 82) guarantees the existence of two spheres $U_{j} = \left\{ {{\mathbf{u}}_{j} |\left\| {{\mathbf{u}}_{j} - {{\boldsymbol{\uplambda}}}_{j}^{*} } \right\| < \delta_{j} } \right\}$ and $V_{j1} = \left\{ {{\mathbf{s}}|\left\| {{\mathbf{s}} - {{\boldsymbol{\upsigma}}}} \right\| < \gamma_{j1} } \right\}$ such that the equation ${\mathbf{F}}_{j} \left( {{\mathbf{u}}_{j} ,{\mathbf{s}}} \right) = {\mathbf{0}}$ has a unique solution ${\mathbf{u}}_{j} \left( {\mathbf{s}} \right) \in U_{j}$ for each ${\mathbf{s}} \in V_{j1}$. Such a solution ${\mathbf{u}}_{j} \left( {\mathbf{s}} \right)$ is an eigenvector of $\, {\mathbf{A}}_{j} \left( {\mathbf{s}} \right)$, but is not necessarily the first one ${{\boldsymbol{\uplambda}}}_{j}^{*} \left( {\mathbf{s}} \right)$. However, continuity of ${{\boldsymbol{\uplambda}}}_{j}^{*} \left( {\mathbf{s}} \right)$ at point ${\mathbf{s}} = {{\boldsymbol{\upsigma}}}$ implies the existence of a neighborhood $V_{j2} = \left\{ {{\mathbf{s}}|\left\| {{\mathbf{s}} - {{\boldsymbol{\upsigma}}}} \right\| < \gamma_{j2} } \right\}$ of ${{\boldsymbol{\upsigma}}}$ such that ${{\boldsymbol{\uplambda}}}_{j}^{*} \left( {\mathbf{s}} \right) \in U_{j}$ for any ${\mathbf{s}} \in V_{j2}$. For ${\mathbf{s}} \in V_{j} = V_{j1} \cap V_{j2}$, three conditions are satisfied: ${{\boldsymbol{\uplambda}}}_{j}^{*} \left( {\mathbf{s}} \right) \in U_{j}$, ${\mathbf{F}}_{j} \left( {{{\boldsymbol{\uplambda}}}_{j}^{*} \left( {\mathbf{s}} \right),{\mathbf{s}}} \right) = {\mathbf{0}}$ and ${\mathbf{F}}_{j} \left( {{\mathbf{u}}_{j} ,{\mathbf{s}}} \right) = {\mathbf{0}}$ has a unique solution ${\mathbf{u}}_{j} \left( {\mathbf{s}} \right) \in U_{j}$. Therefore, we can conclude that ${{\boldsymbol{\uplambda}}}_{j}^{*} \left( {\mathbf{s}} \right)$ is precisely the unique point in $U_{j}$ verifying ${\mathbf{F}}_{j} \left( {{\mathbf{u}}_{j} ,{\mathbf{s}}} \right) = {\mathbf{0}}$ for ${\mathbf{s}} \in V_{j}$. So, ${{\boldsymbol{\uplambda}}}_{j}^{*} \left( {\mathbf{s}} \right):V_{j} \to U_{j}$ is the unique mapping verifying ${\mathbf{F}}_{j} \left( {{{\boldsymbol{\uplambda}}}_{j}^{*} \left( {\mathbf{s}} \right),{\mathbf{s}}} \right) = {\mathbf{0}}$. Thus, for any ${\mathbf{s}} \in V_{j}$, ${{\boldsymbol{\uplambda}}}_{j}^{*} \left( {\mathbf{s}} \right)$ is closer to ${{\boldsymbol{\uplambda}}}_{j}^{*}$ than any other eigenvector of $\, {\mathbf{A}}_{j} \left( {\mathbf{s}} \right)$.

**Proof of Point b:**

According to the implicit function theorem, the mapping ${{\boldsymbol{\uplambda}}}_{j}^{*} \left( {\mathbf{s}} \right):V_{j} \to U_{j}$ is continuously differentiable and its derivative is given by the formula

A2 $$\frac{{\partial {{\boldsymbol{\uplambda}}}_{j}^{*} \left( {\mathbf{s}} \right)}}{{\partial {\mathbf{s}}^{t} }} = - \left[ {\frac{{\partial {\mathbf{F}}_{j} }}{{\partial {\mathbf{u}}_{j}^{t} }}\left( {{{\boldsymbol{\uplambda}}}_{j}^{*} \left( {\mathbf{s}} \right),{\mathbf{s}}} \right)} \right]^{ - 1} \frac{{\partial {\mathbf{F}}_{j} }}{{\partial {\mathbf{s}}^{t} }}\left( {{{\boldsymbol{\uplambda}}}_{j}^{*} \left( {\mathbf{s}} \right),{\mathbf{s}}} \right).$$

From ${\mathbf{f}}_{j} \left( {{\mathbf{u}}_{j} ,{{\boldsymbol{\upsigma}}}} \right) = sign\left( {{{\boldsymbol{\uplambda}}}_{j}^{t} {\mathbf{u}}_{j} } \right)\frac{{{{\boldsymbol{\uplambda}}}_{j} }}{{\left\| {{{\boldsymbol{\uplambda}}}_{j} } \right\|}}$ we obtain $\frac{{\partial {\mathbf{F}}_{j} }}{{\partial {\mathbf{u}}_{j}^{t} }}\left( {{{\boldsymbol{\uplambda}}}_{j}^{*} ,{{\boldsymbol{\upsigma}}}} \right) = {\mathbf{I}}_{{p_{j} }}$. Formula (A1) is then deduced from this result and $\frac{{\partial {\mathbf{F}}_{j} }}{{\partial {\mathbf{s}}^{t} }}\left( {{\mathbf{u}}_{j} ,{\mathbf{s}}} \right) = - \frac{{\partial {\mathbf{f}}_{j} }}{{\partial {\mathbf{s}}^{t} }}\left( {{\mathbf{u}}_{j} ,{\mathbf{s}}} \right)$.

The statistical properties of ${{\boldsymbol{\uplambda}}}_{j}^{*} \left( {\mathbf{s}} \right)$ and of any smooth function ${\mathbf{g}}\left( {{{\boldsymbol{\uplambda}}}_{1}^{*} \left( {\mathbf{s}} \right),...,{{\boldsymbol{\uplambda}}}_{J}^{*} \left( {\mathbf{s}} \right);{\mathbf{s}}} \right)$ are now given in the next proposition.

### Proposition 2

Assuming the specific measurement model studied in this paper, the following properties hold:

- (**a**) ${{\boldsymbol{\uplambda}}}_{j}^{*} \left( {\mathbf{s}} \right)$ is a CAN estimator of ${{\boldsymbol{\uplambda}}}_{j}^{*}$:
  A3 $${{\boldsymbol{\uplambda}}}_{j}^{*} \left( {\mathbf{s}} \right) \sim AN\left( {{{\boldsymbol{\uplambda}}}_{j}^{*} ,\frac{{\partial {\mathbf{f}}_{j} }}{{\partial {\mathbf{s}}^{t} }}\left( {{{\boldsymbol{\uplambda}}}_{j}^{*} ,{{\boldsymbol{\upsigma}}}} \right)var\left( {\mathbf{s}} \right)\frac{{\partial {\mathbf{f}}_{j} }}{{\partial {\mathbf{s}}}}\left( {{{\boldsymbol{\uplambda}}}_{j}^{*} ,{{\boldsymbol{\upsigma}}}} \right)} \right) .$$
- **(b) **${\mathbf{g}}\left( {{{\boldsymbol{\uplambda}}}_{1}^{*} \left( {\mathbf{s}} \right),...,{{\boldsymbol{\uplambda}}}_{J}^{*} \left( {\mathbf{s}} \right),{\mathbf{s}}} \right)$ is a CAN estimator of ${\mathbf{g}}\left( {{{\boldsymbol{\uplambda}}}_{1}^{*} ,...,{{\boldsymbol{\uplambda}}}_{J}^{*} ,{{\boldsymbol{\upsigma}}}} \right)$ for any smooth function **g**:
  A4 $$\begin{gathered} {\mathbf{g}}\left( {{{\boldsymbol{\uplambda}}}_{1}^{*} \left( {\mathbf{s}} \right),...,{{\boldsymbol{\uplambda}}}_{J}^{*} \left( {\mathbf{s}} \right),{\mathbf{s}}} \right) \hfill \\ \, \sim AN\left( {{\mathbf{g}}\left( {{{\boldsymbol{\uplambda}}}_{1}^{*} ,...,{{\boldsymbol{\uplambda}}}_{J}^{*} ,{{\boldsymbol{\upsigma}}}} \right),\frac{{{{\rm{d}}}{\mathbf{g}}}}{{{{\rm{d}}}{\mathbf{s}}^{t} }}\left( {{{\boldsymbol{\uplambda}}}_{1}^{*} ,...,{{\boldsymbol{\uplambda}}}_{J}^{*} ,{{\boldsymbol{\upsigma}}}} \right)var\left( {\mathbf{s}} \right)\frac{{{{\rm{d}}}{\mathbf{g}}}}{{{{\rm{d}}}{\mathbf{s}}}}\left( {{{\boldsymbol{\uplambda}}}_{1}^{*} ,...,{{\boldsymbol{\uplambda}}}_{J}^{*} ,{{\boldsymbol{\upsigma}}}} \right)} \right). \hfill \\ \end{gathered}$$

### Proof

Using the property ${\mathbf{s}} \sim AN\left( {{{\boldsymbol{\upsigma}}},var\left( {\mathbf{s}} \right)} \right)$ and the delta method lead to the results.
